# Supplementary material for: Non-Toxicological Role of Aryl Hydrocarbon Receptor in Obesity-Associated Multiple Myeloma Cell Growth and Survival
Source: Cancers (Basel). 2023 Nov 1;15(21):5255. doi: 10.3390/cancers15215255 (PMC10649826; doi:10.3390/cancers15215255)
Supplement: Supplementary file 1 [file cancers-15-05255-s001.zip › supplementary material/cancers-2634977-Original Western Blot.pdf]

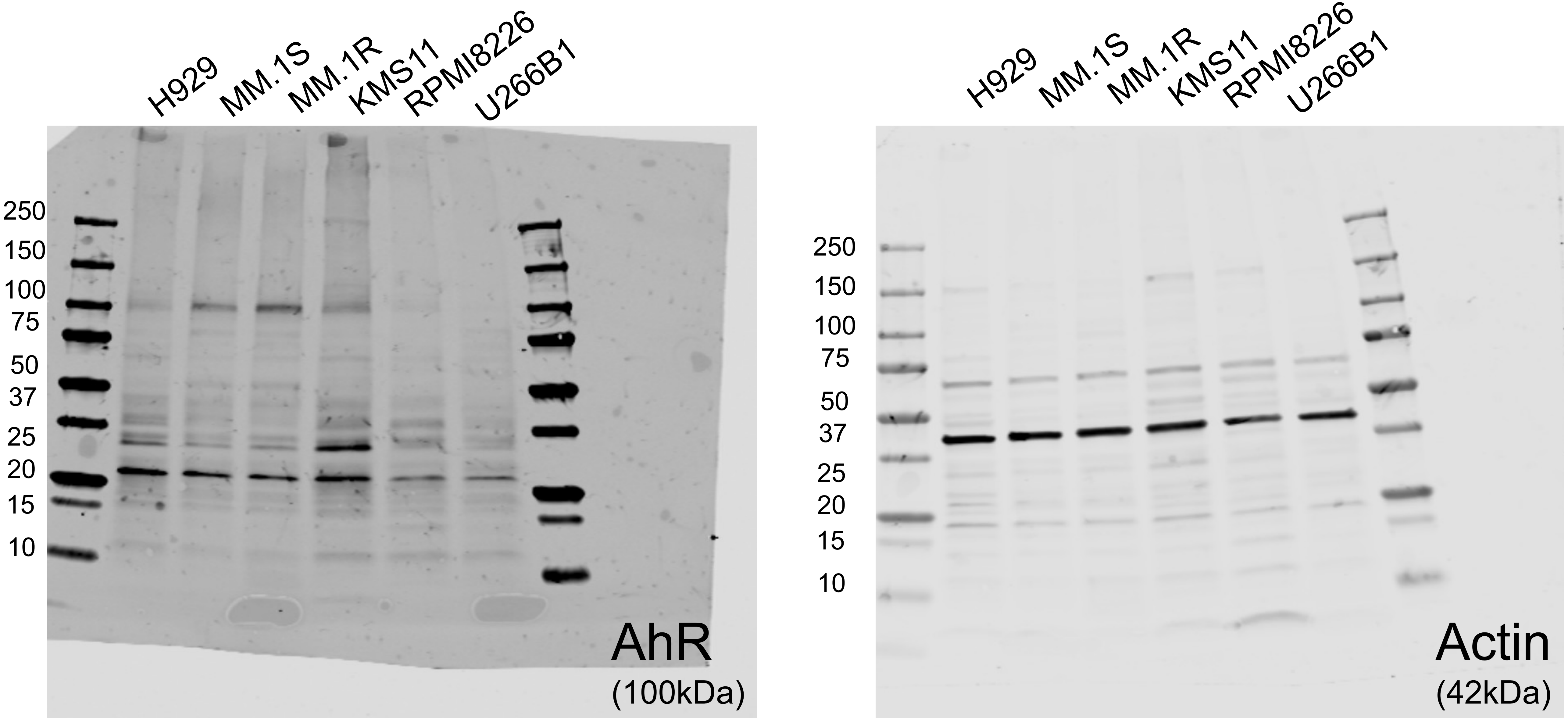
H929
MM.1S
MM.1R
KMS11
RPMI8226
U266B1
250
150
100
75
50
37
25
20
15
10
AhR
(100kDa)
H929
MM.1S
MM.1R
KMS11
RPMI8226
U266B1
250
150
100
75
50
37
25
20
15
10
Actin
(42kDa)

Figure 1A

Figure 1B –

MM.1S Agonists/Antagonists

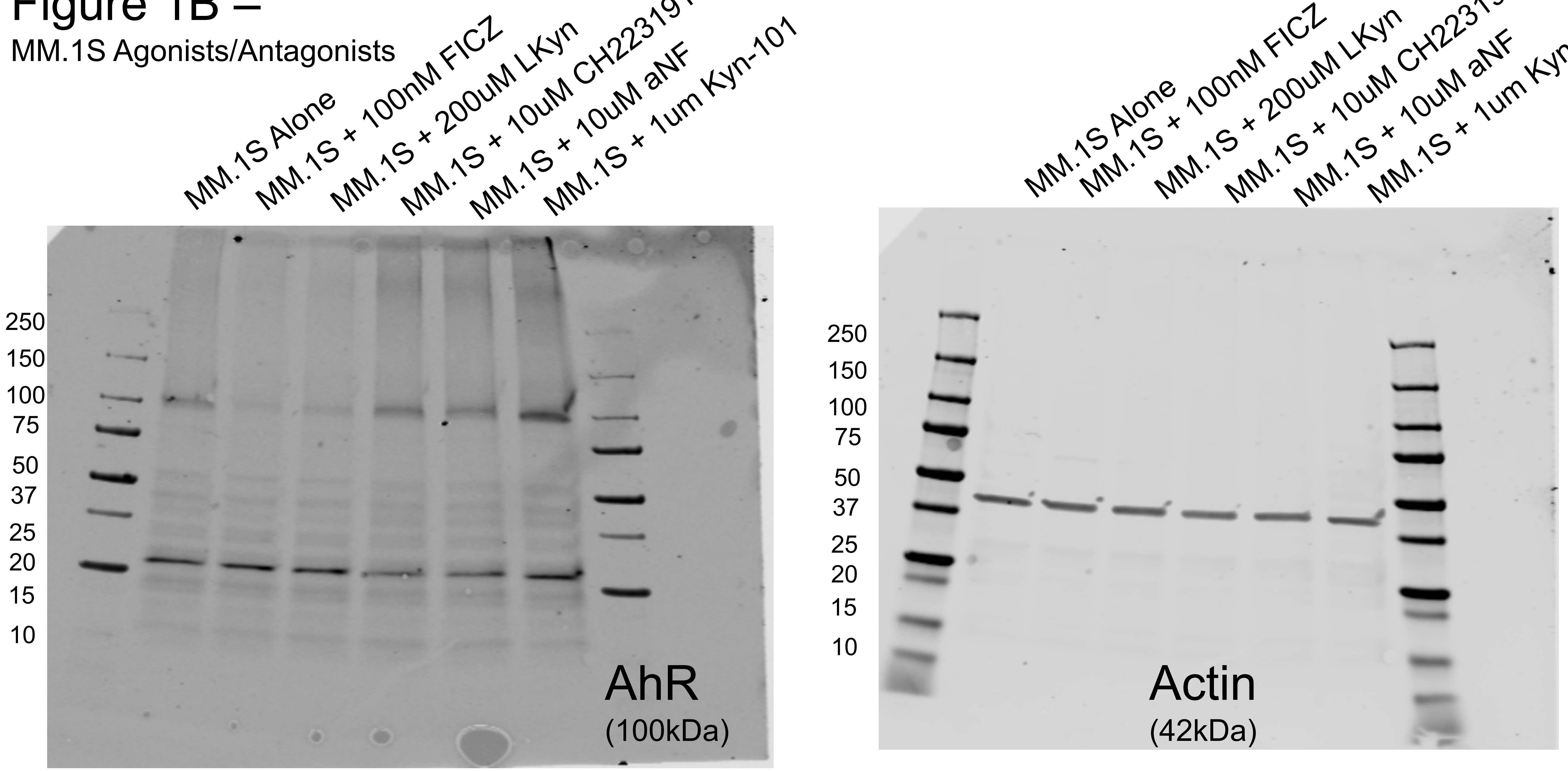

Figure 1B –

MM.1R Agonists/Antagonists

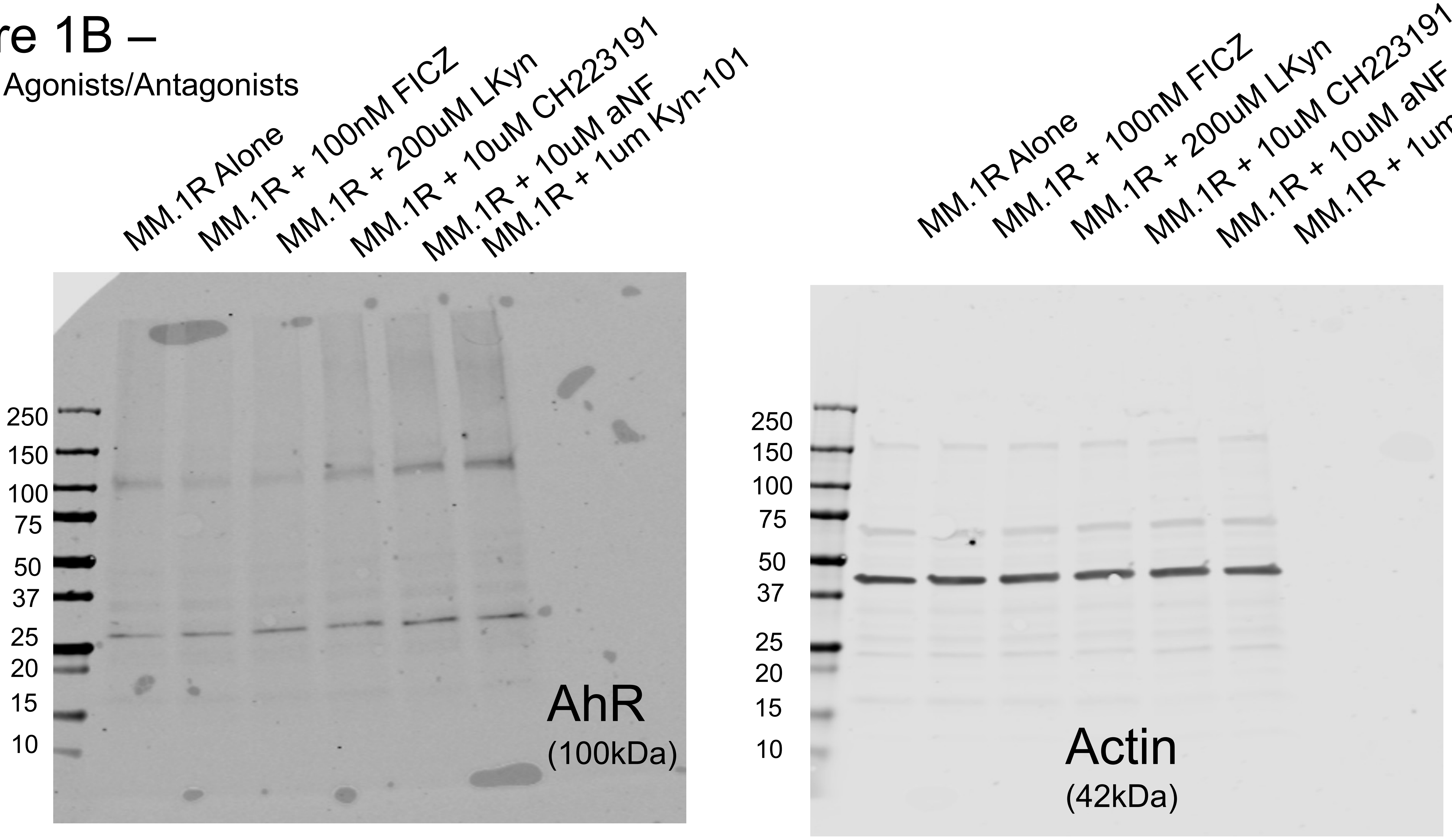

Figure 1B –

KMS11 Agonists/Antagonists

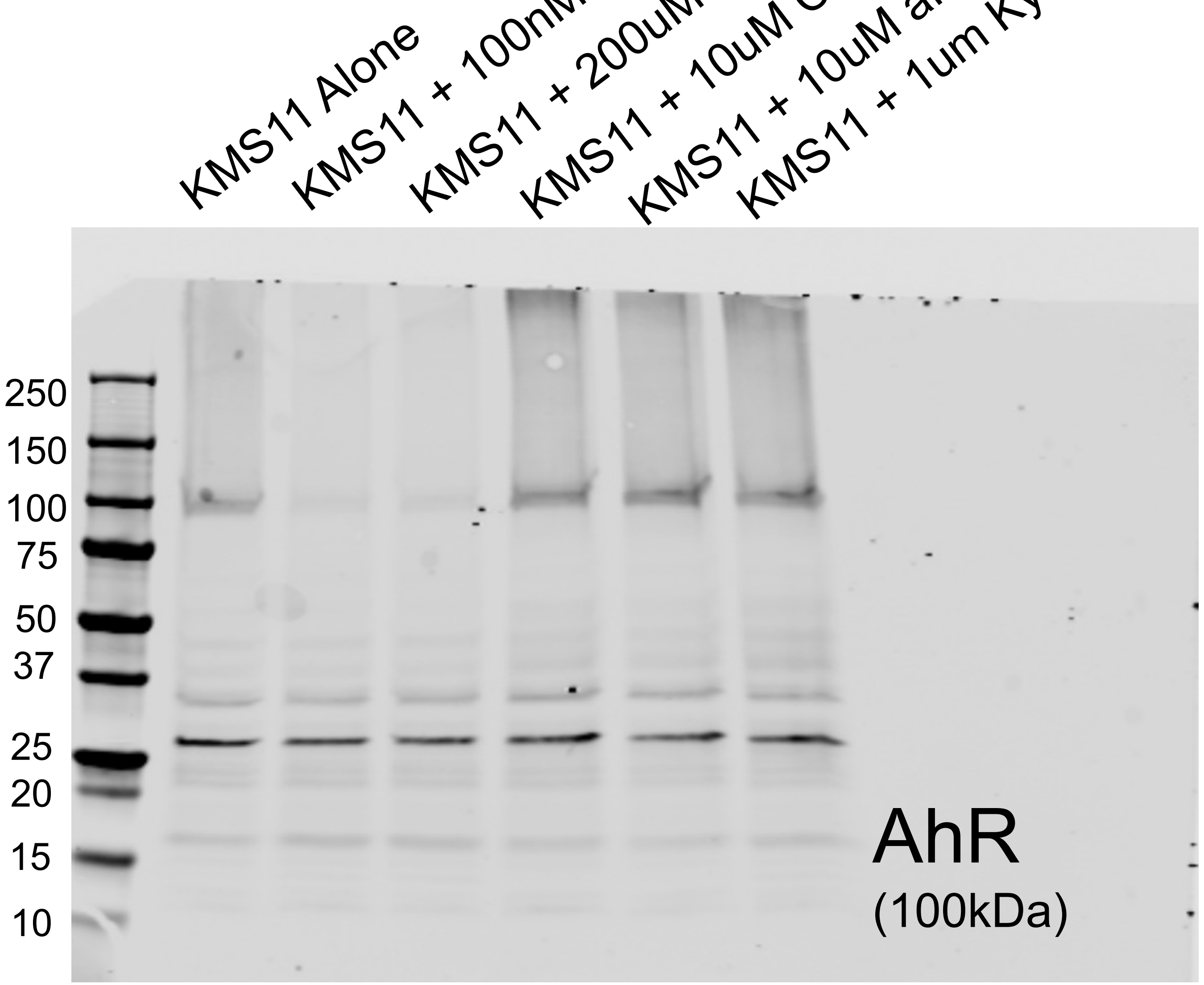

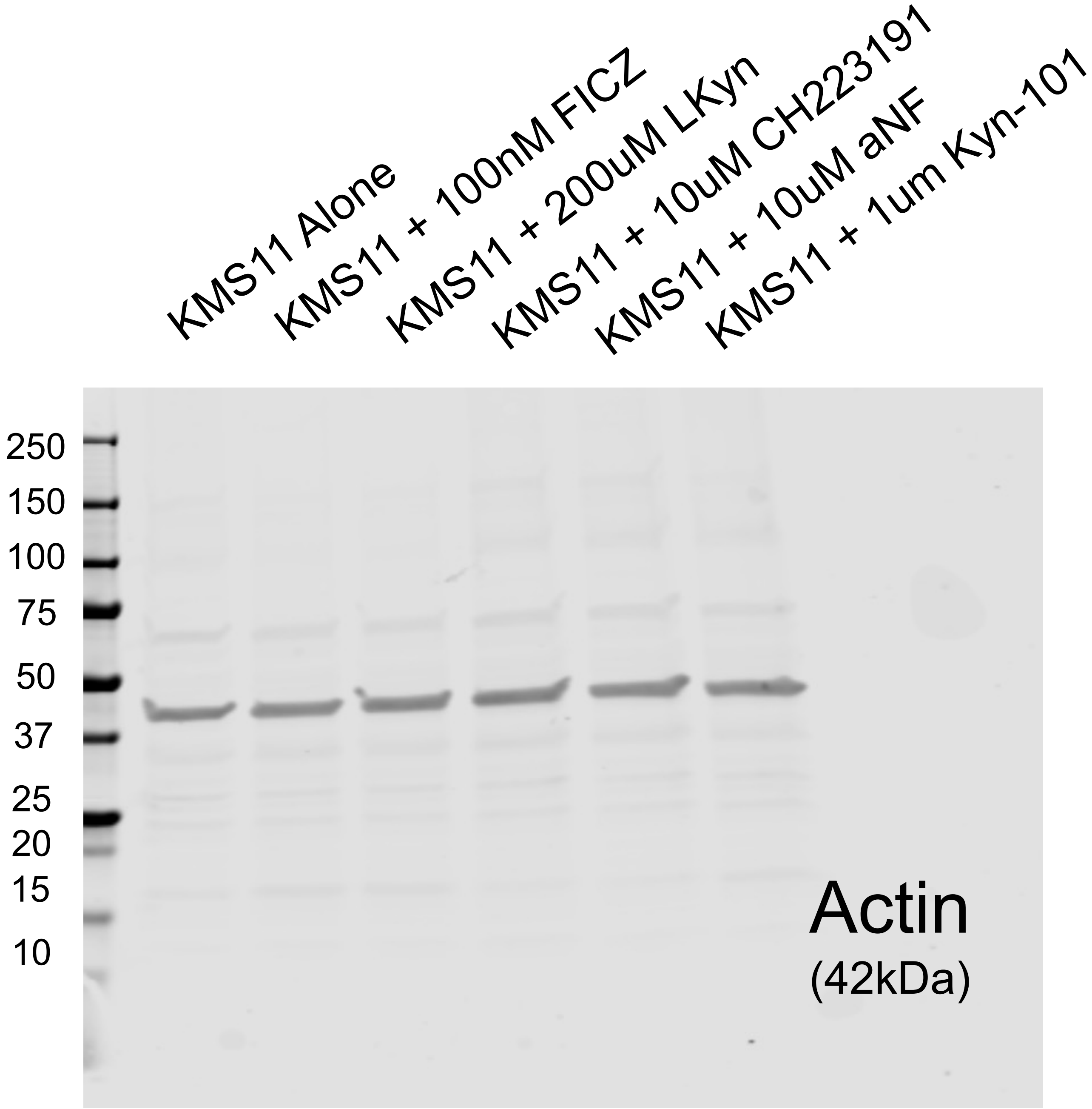

Figure 1B –

H929 Agonists/Antagonists

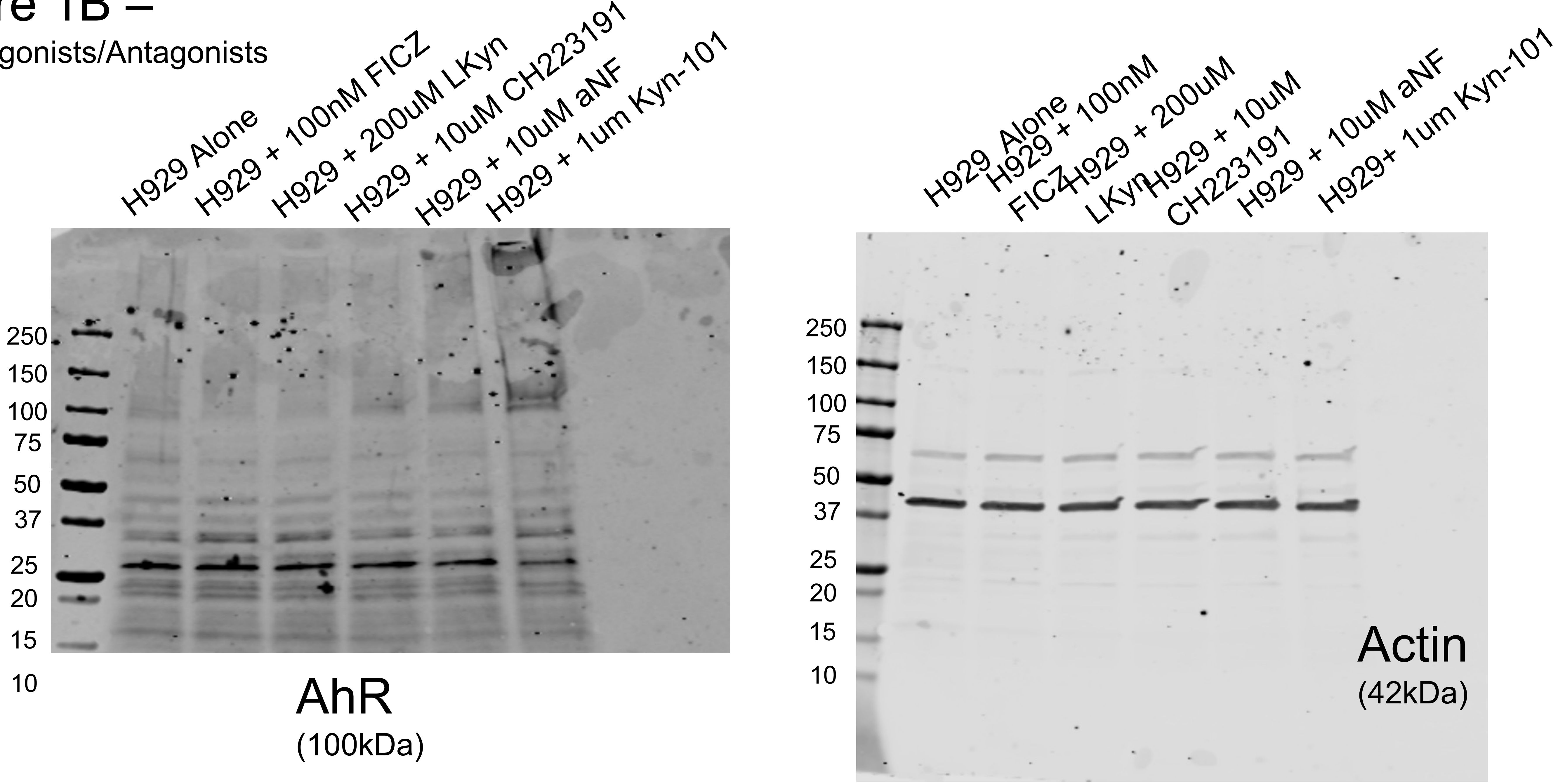

Figure 1B –
RPMI8226
Agonists/Antagonists

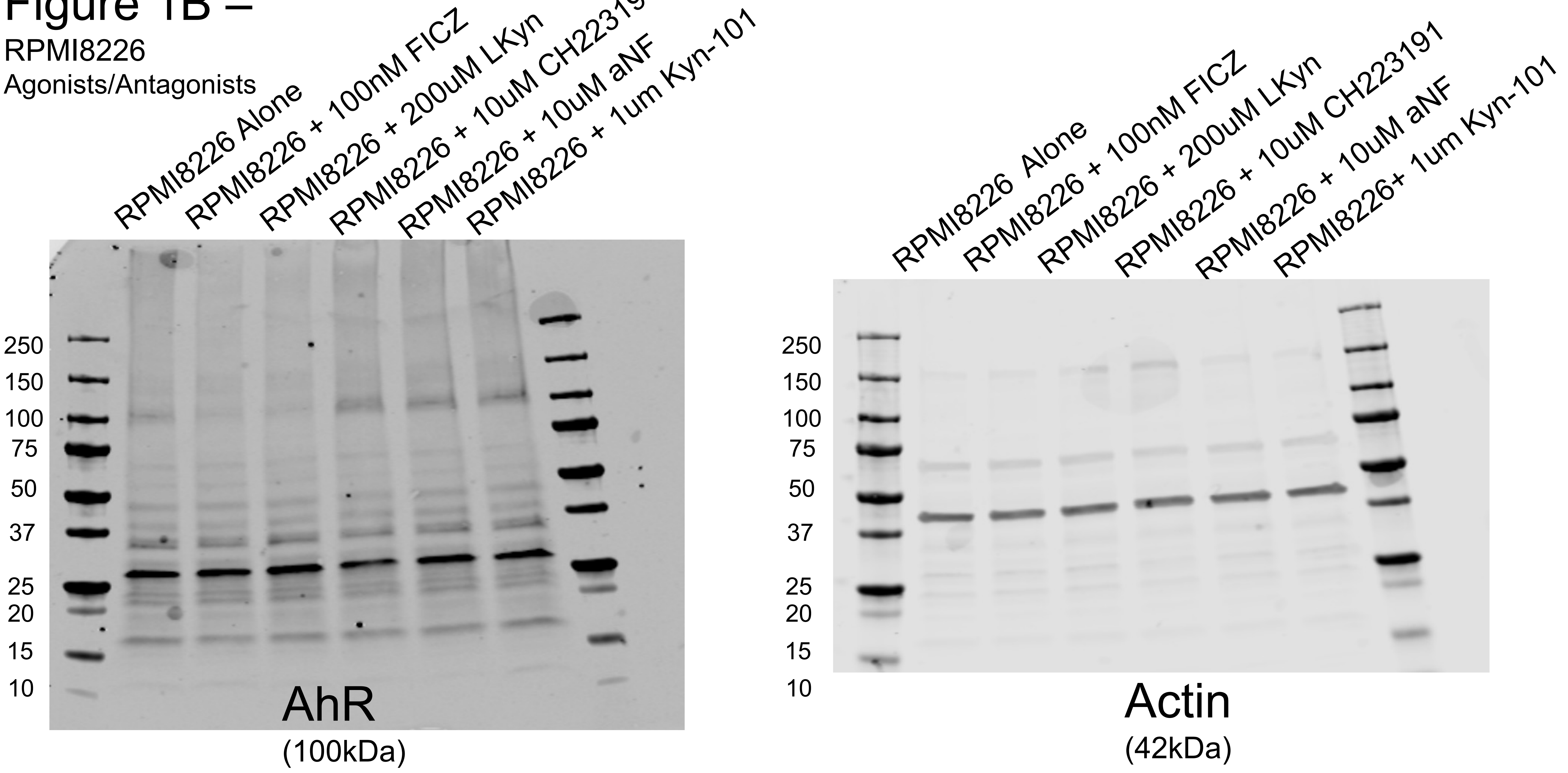

Figure 1B –

U266B1 Agonists/Antagonists

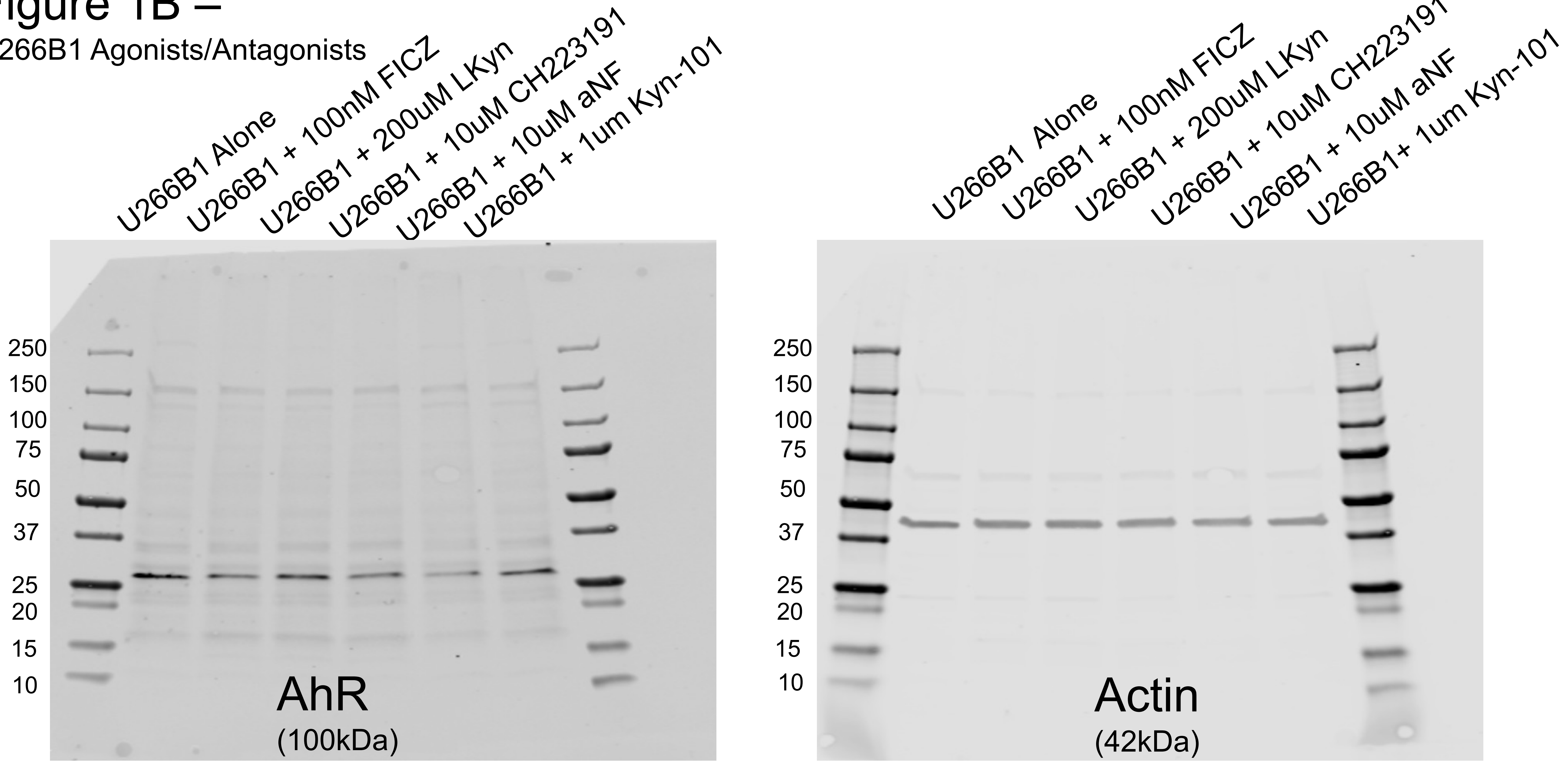

Figure 1C –

MM.1S Agonists/Antagonists Fractionations

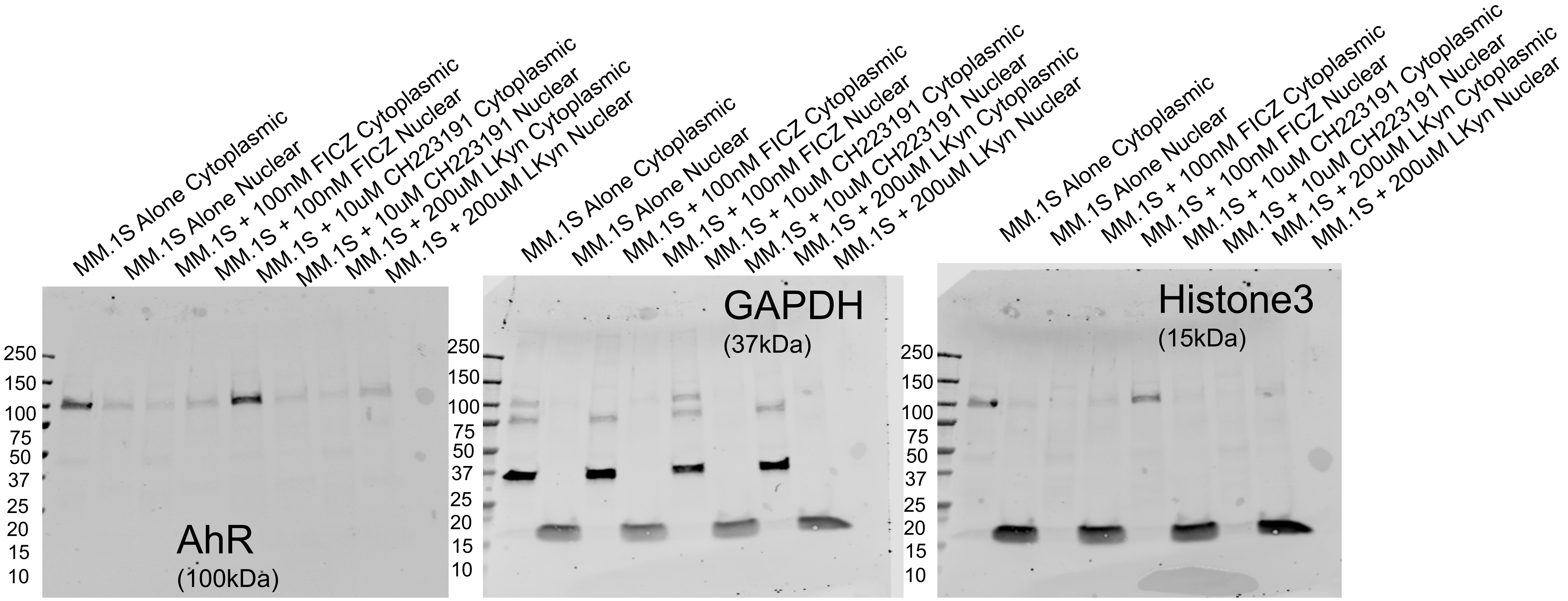

Figure 1C –

KMS11 Agonists/Antagonists Fractionations

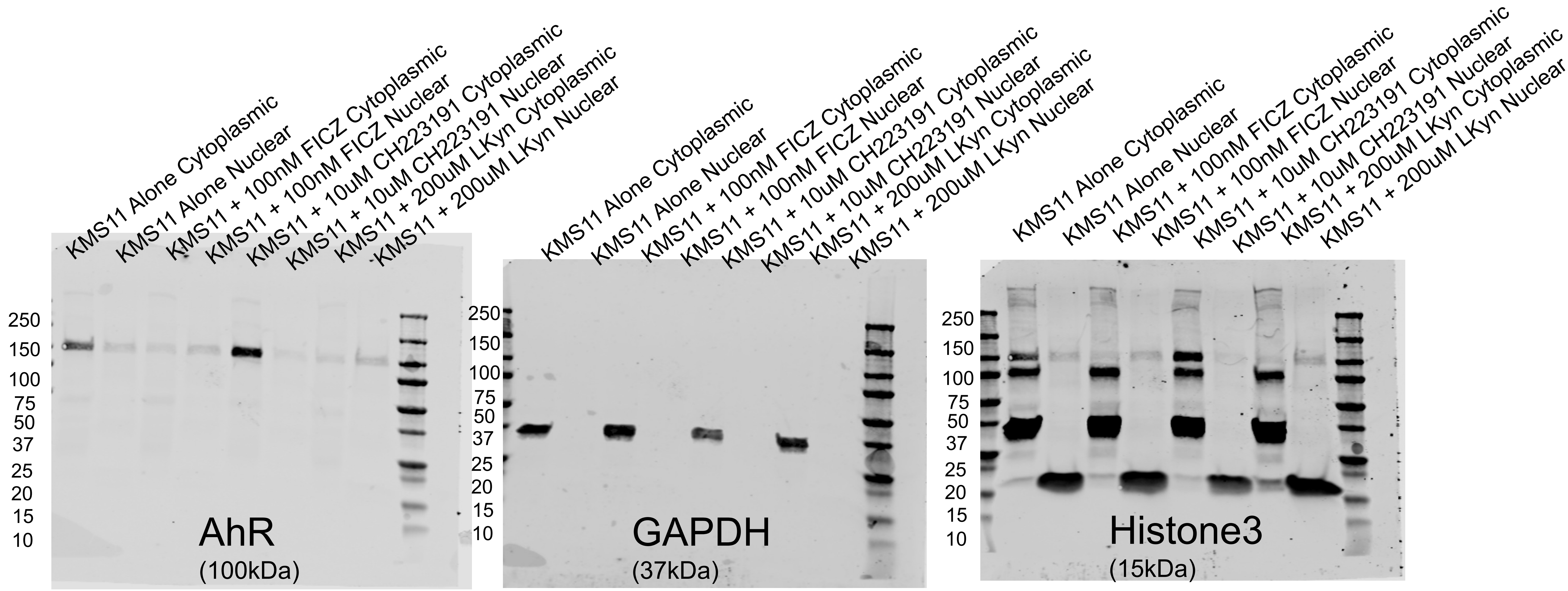

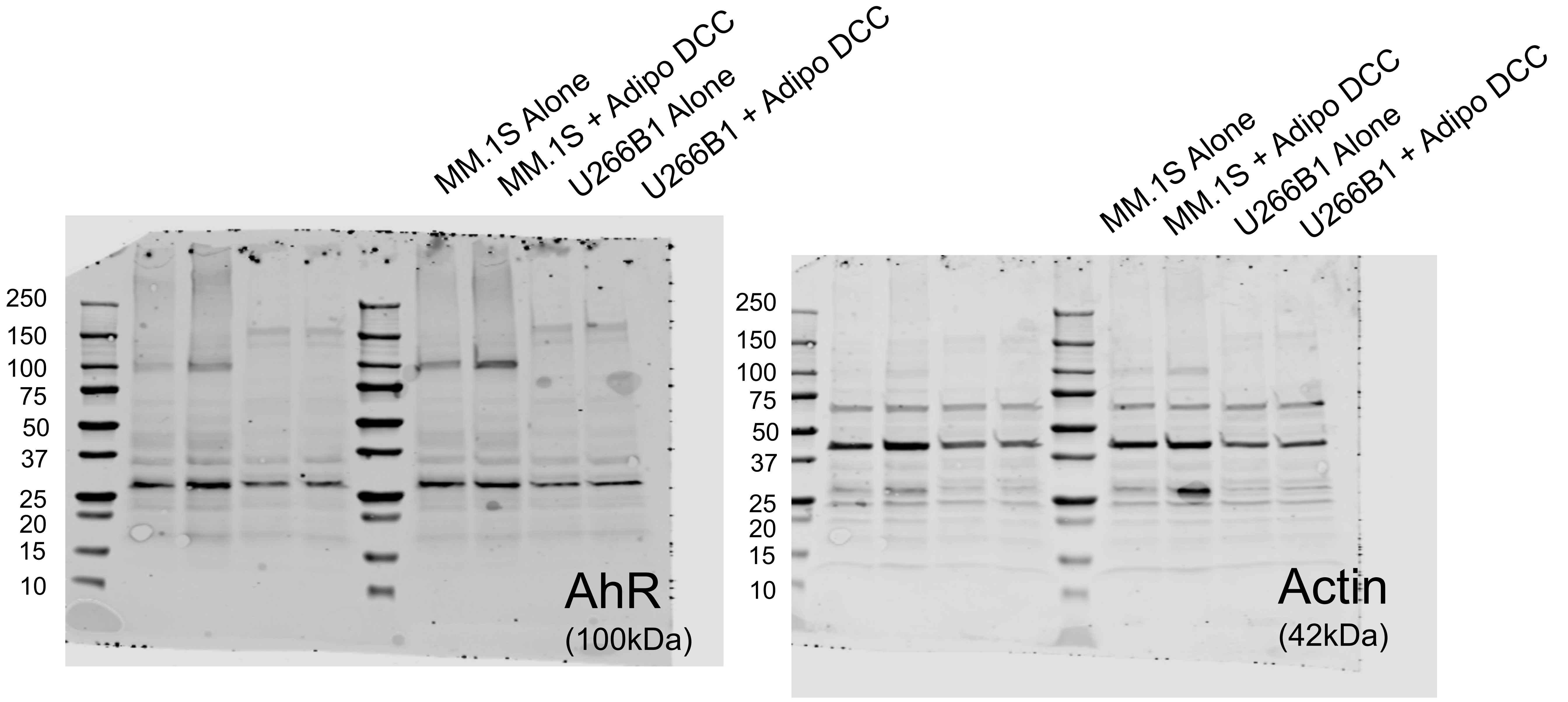

Figure 3B – MM.1S and U266B1 Alone vs Adipo DCC

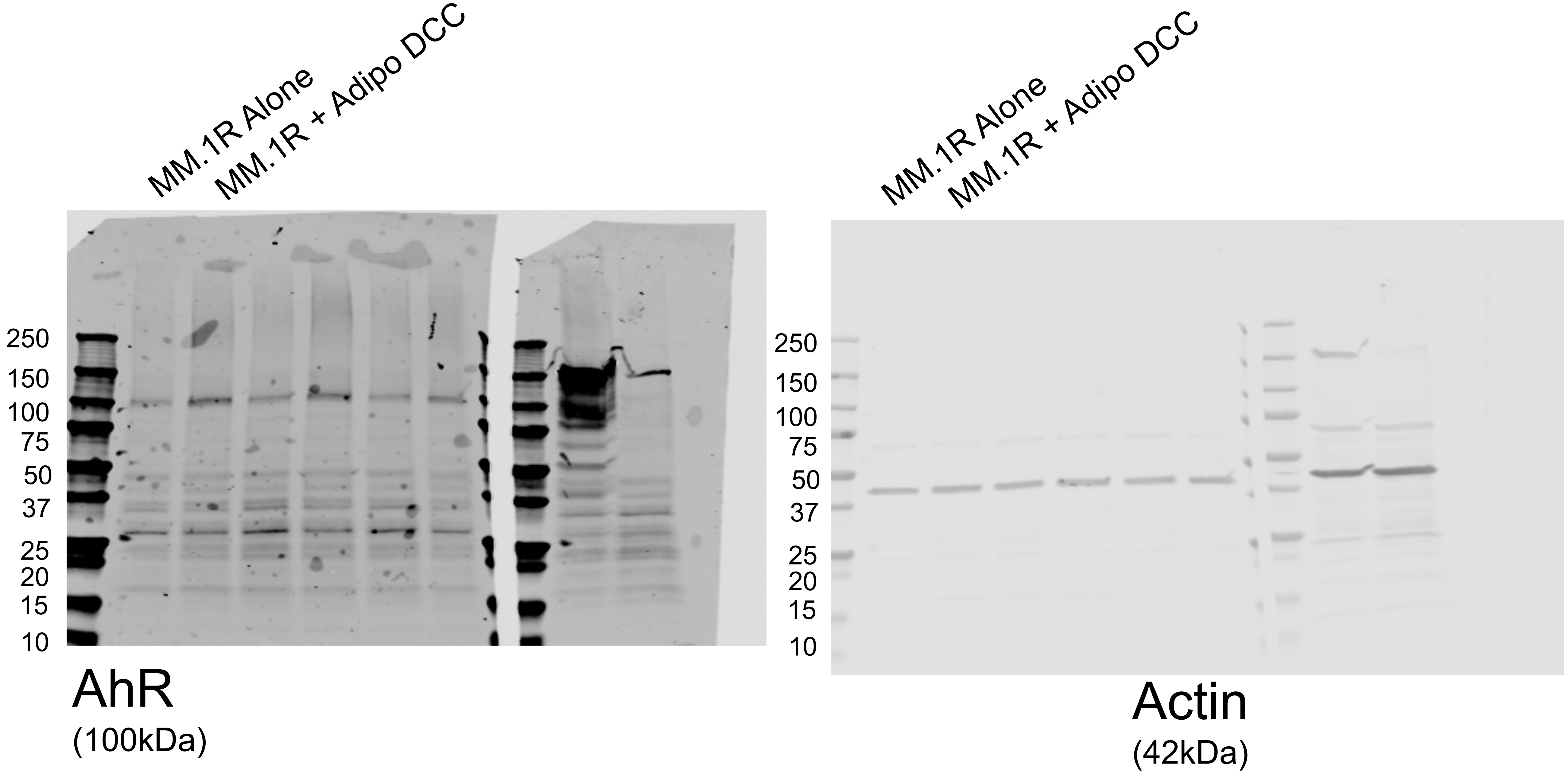

Figure 3B – MM.1R Alone vs Adipo DCC

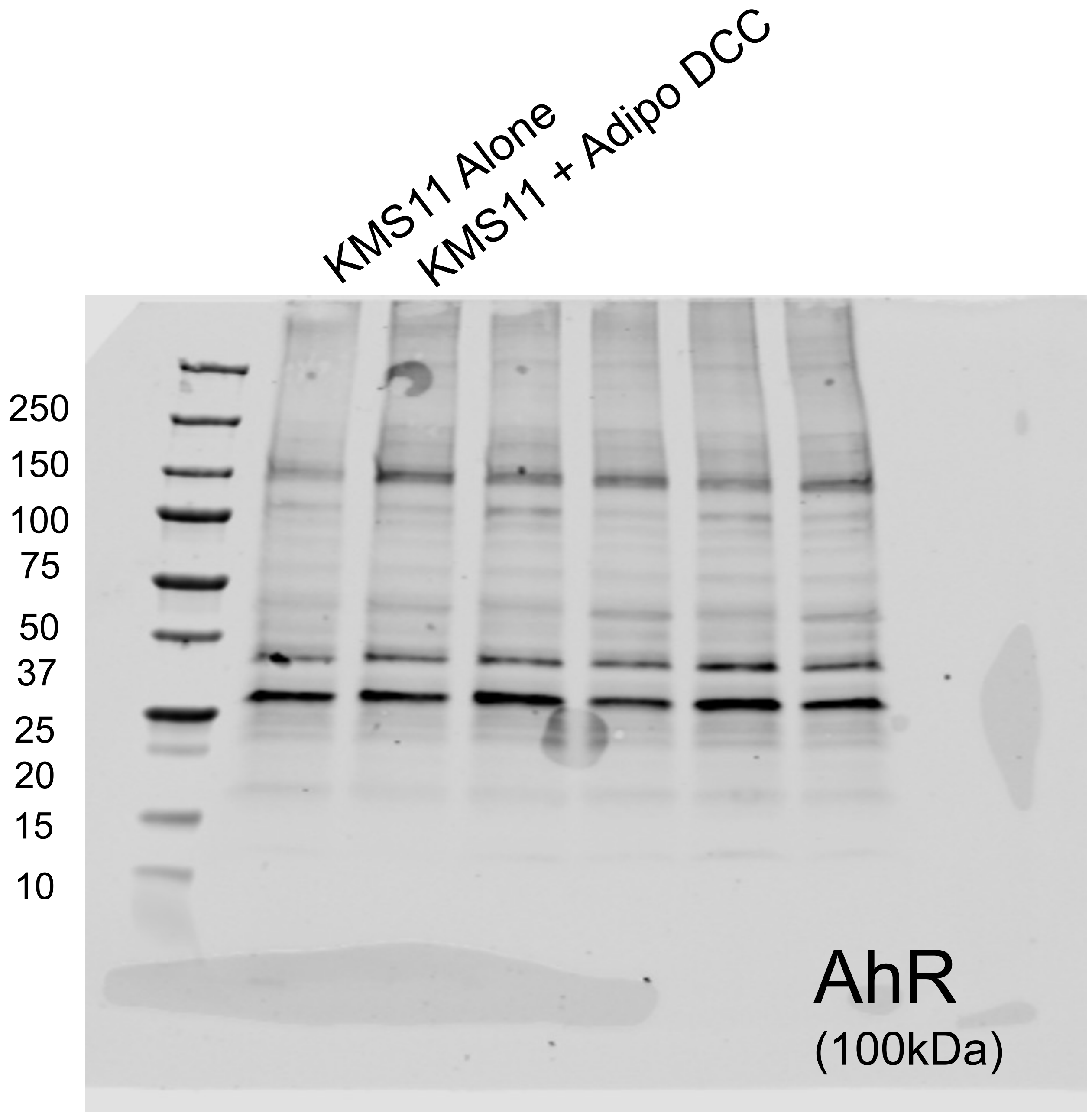

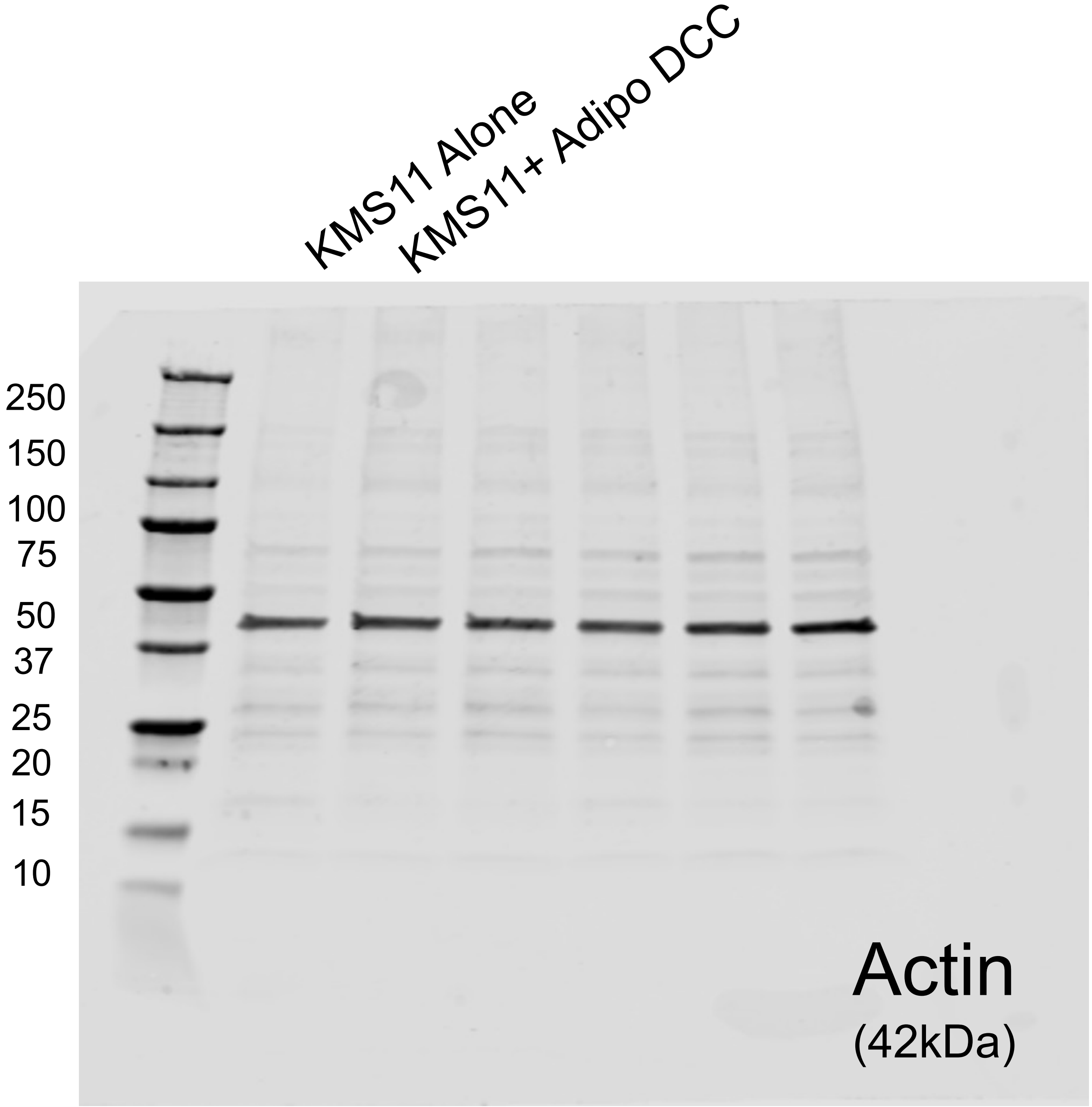

Figure 3B – KMS11 Alone vs Adipo DCC

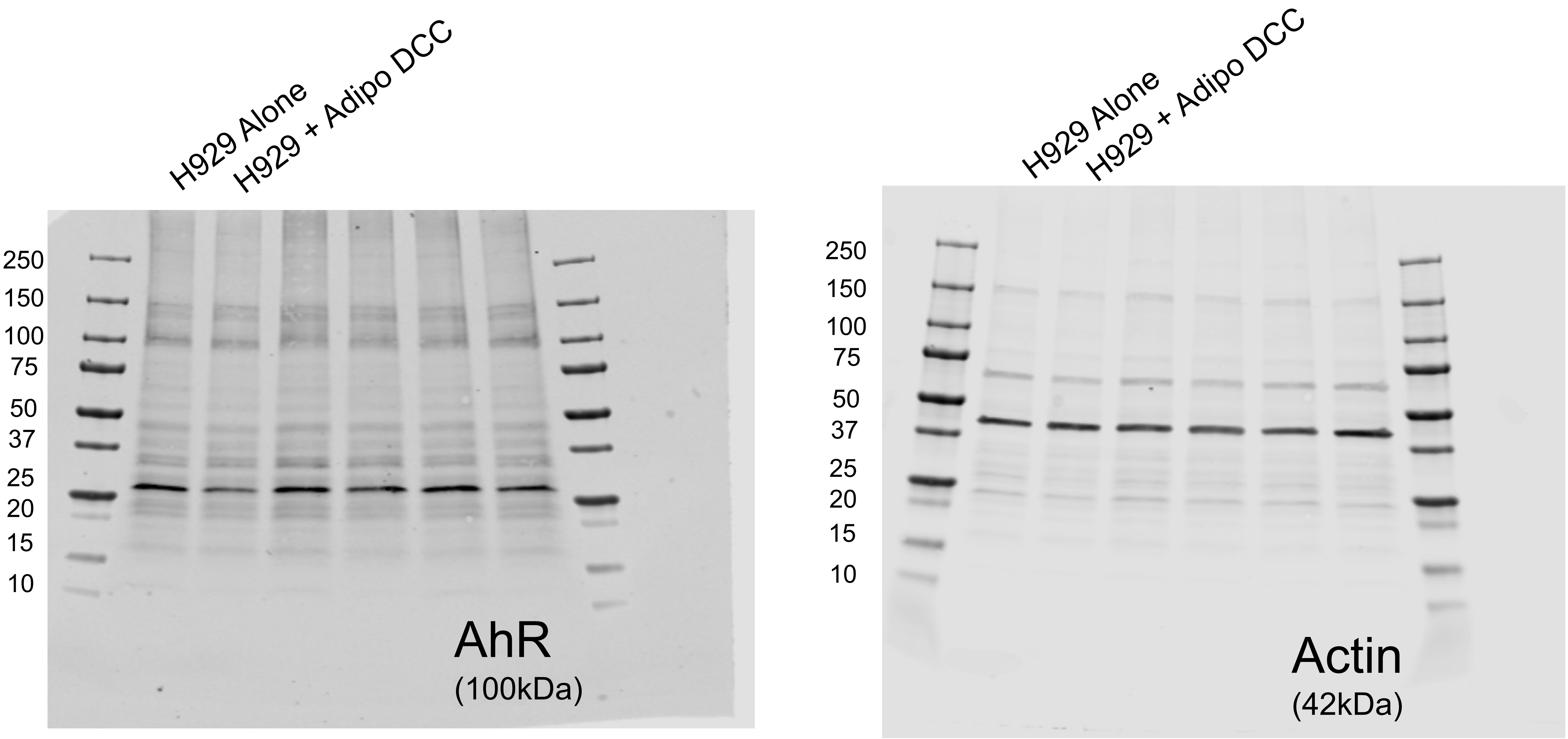

Figure 3B – H929 Alone vs Adipo DCC

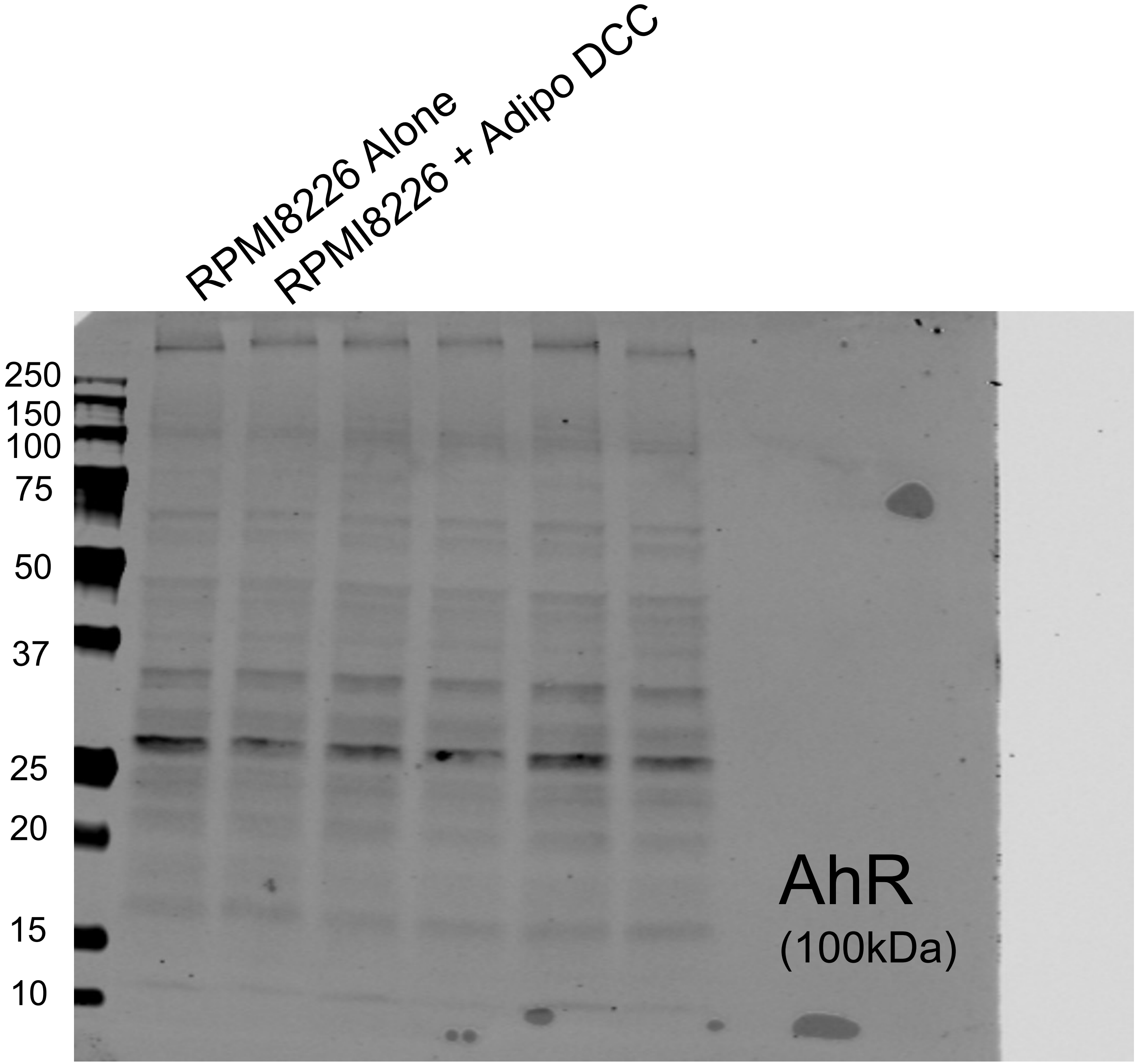

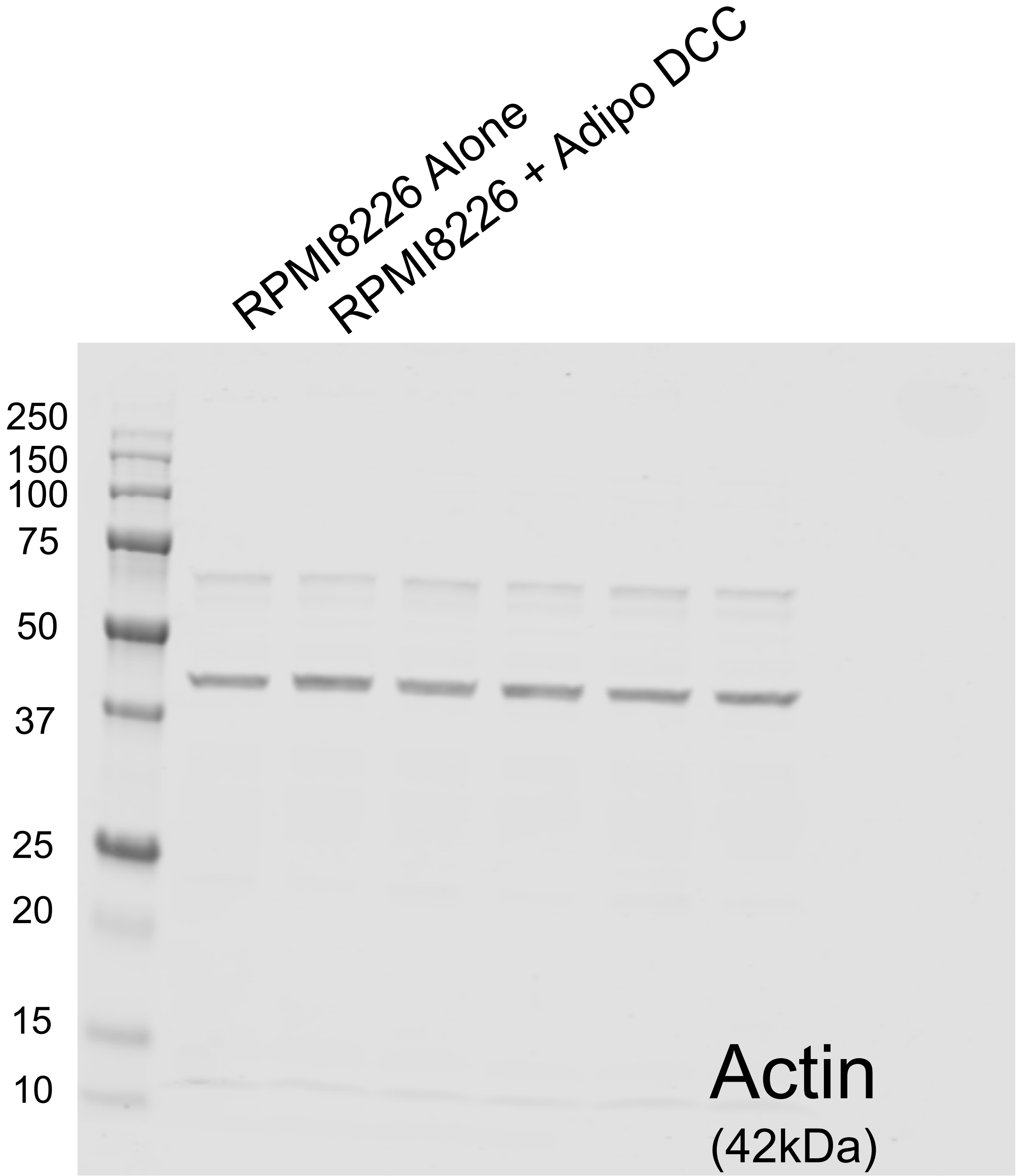

Figure 3B – RPMI8226 Alone vs Adipo DCC

Figure 3C –

MM.1S Adipo DCC Fractionations

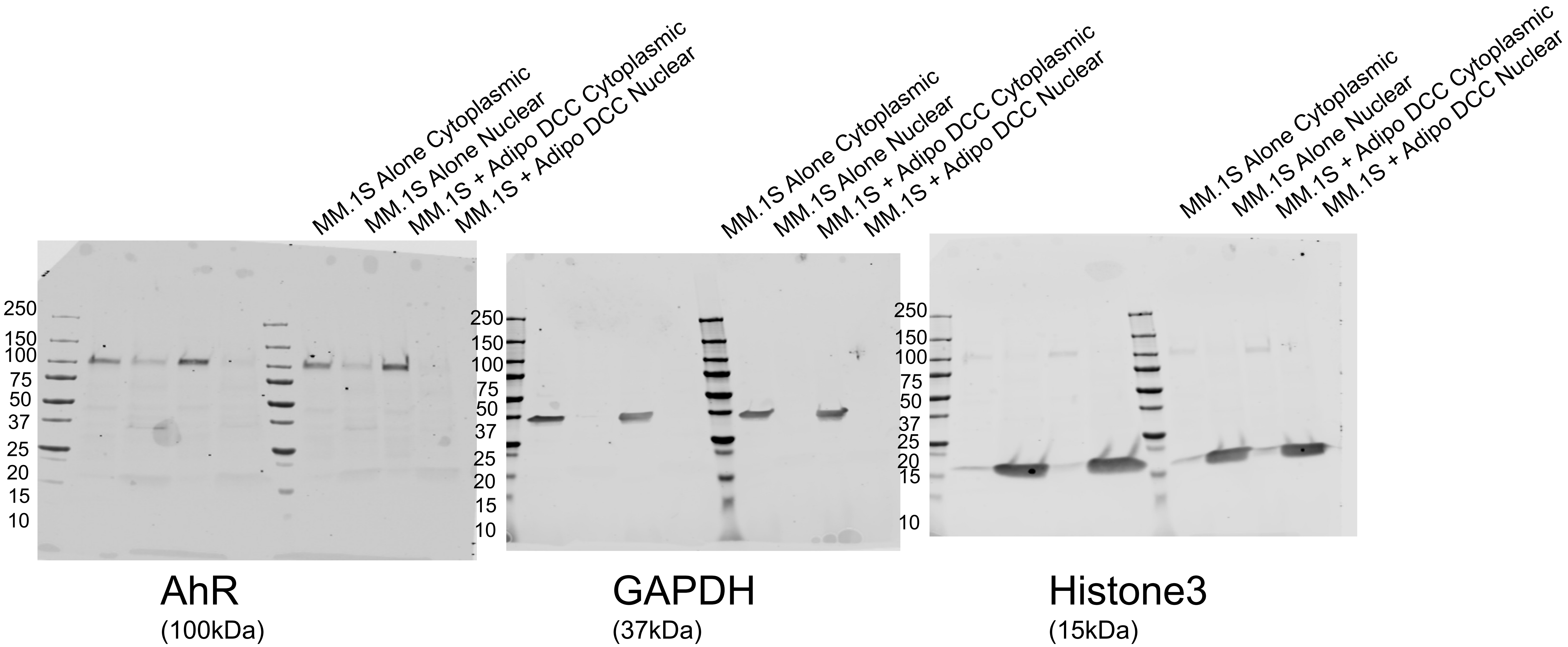

Figure 3C –

KMS11 Adipo DCC Fractionations

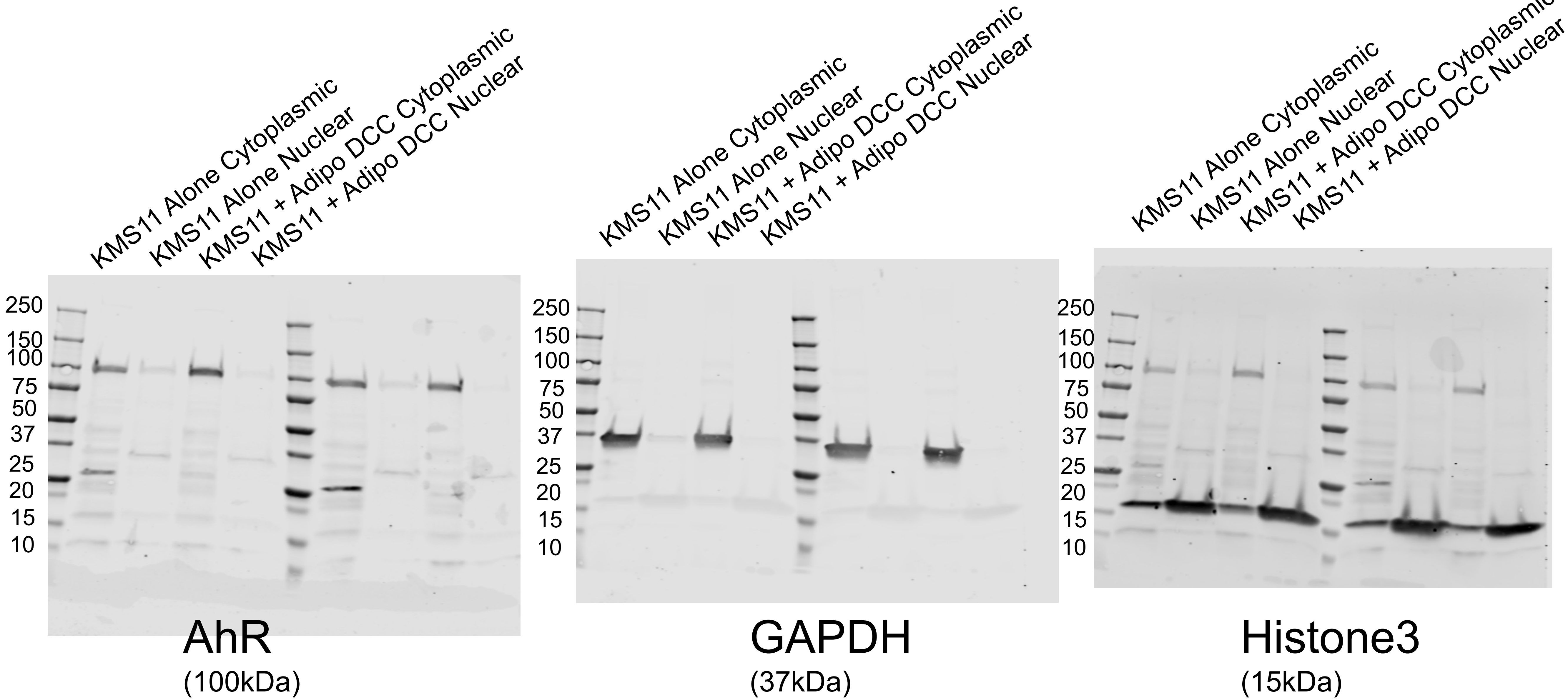

Figure 3C –

H929 and RPMI8226 Adipo DCC Fractionations

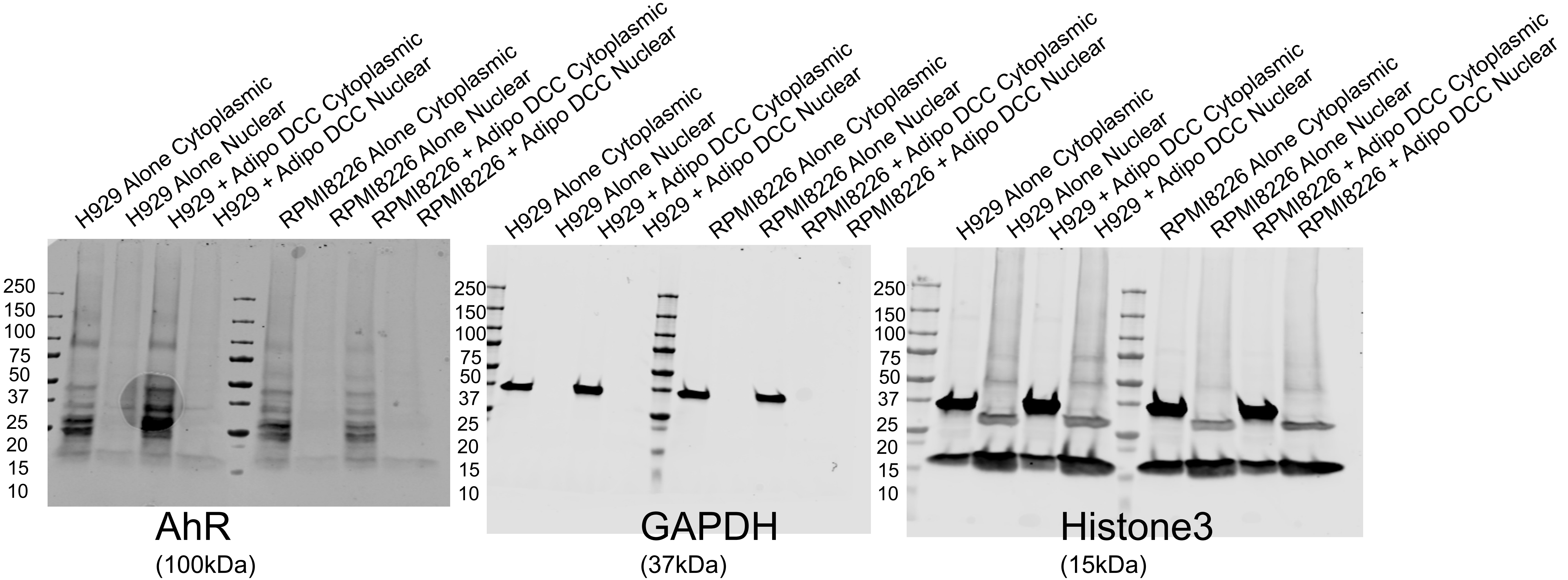

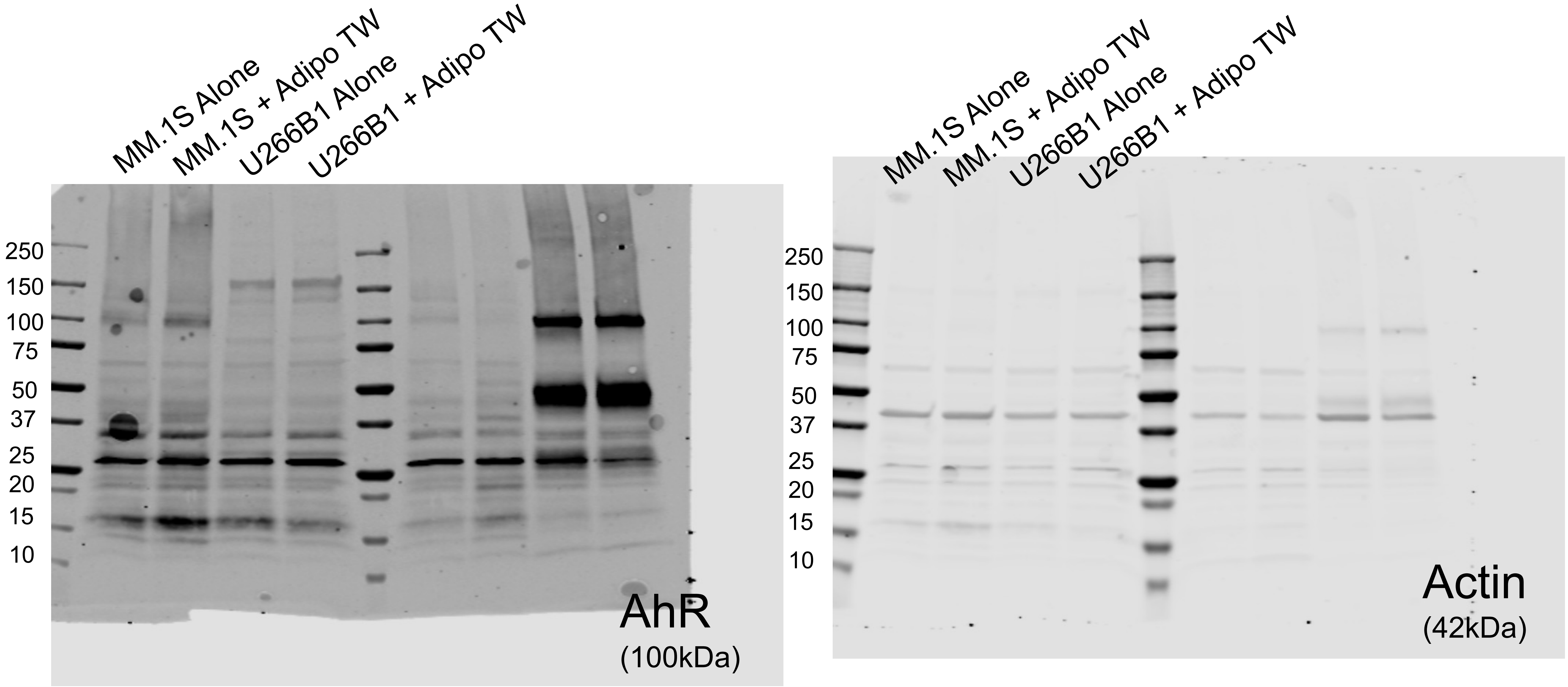

Figure 3E – MM.1S and U266B1 Alone/Adipo TW

Figure 4A –
MM.1S Alone/Adipo DCC +/- Kynurenine

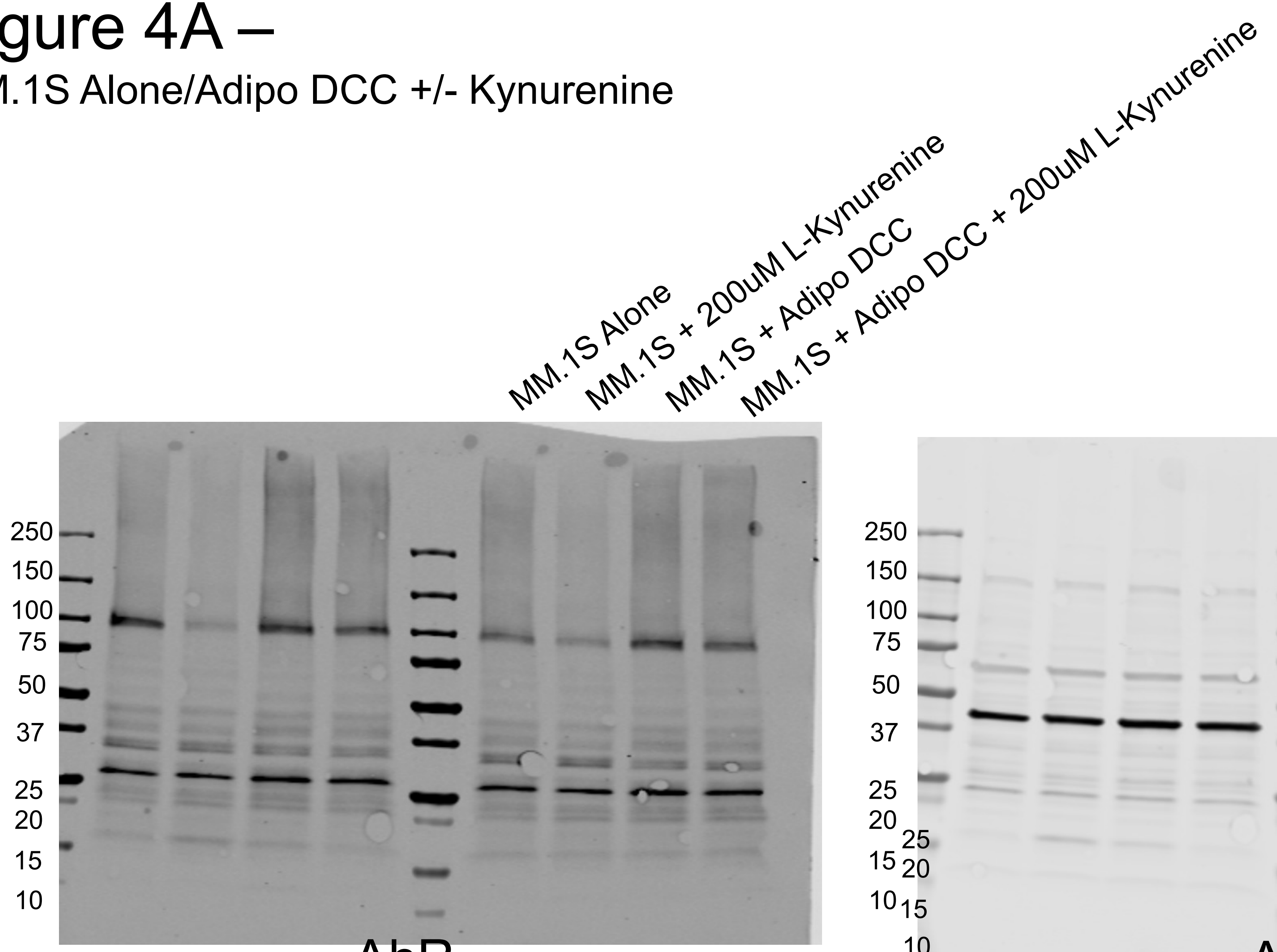

AhR
(100kDa)

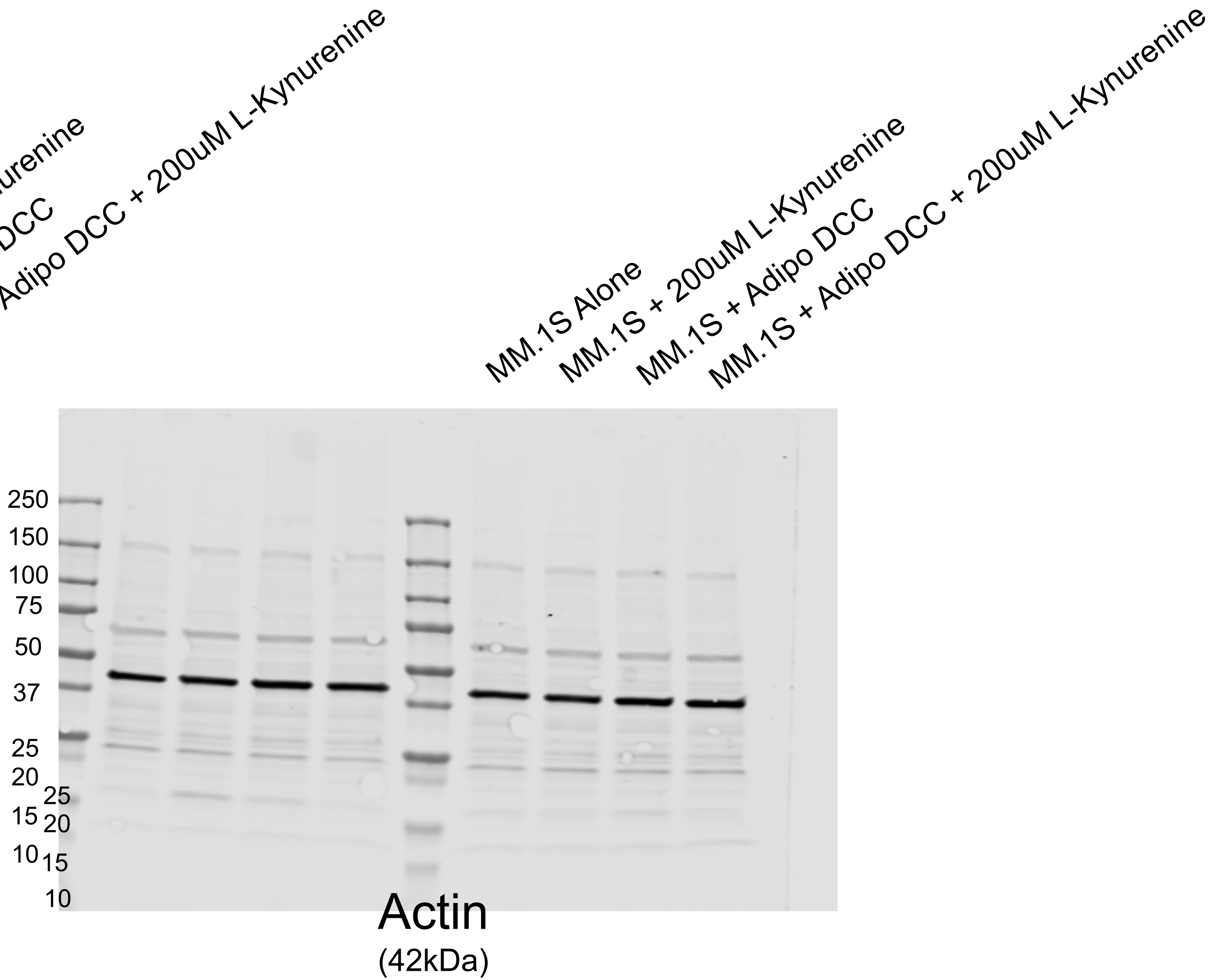

Actin
(42kDa)

Figure 4E –

MM.1S Alone/Adipo DCC +/- 100nM FICZ

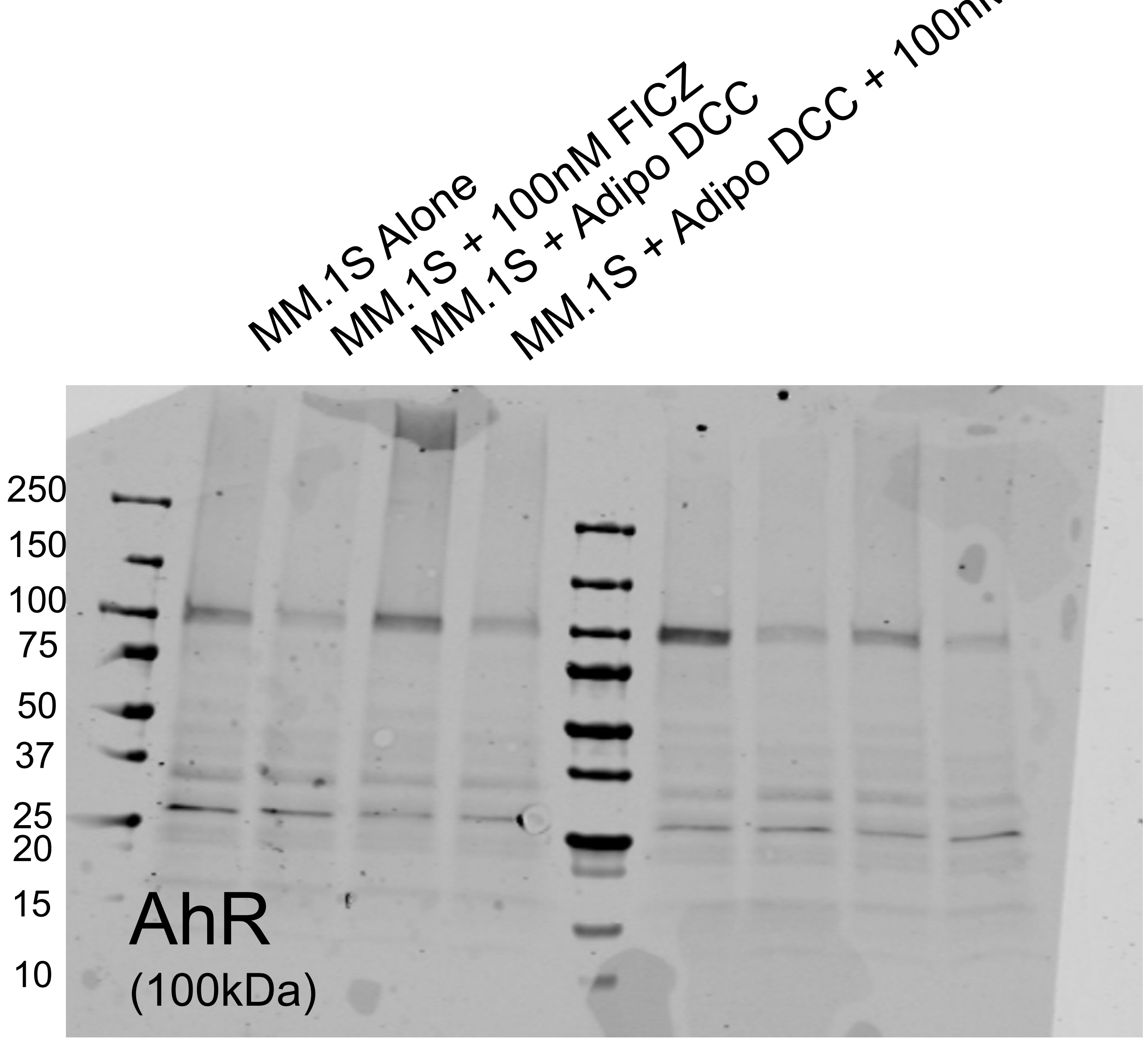

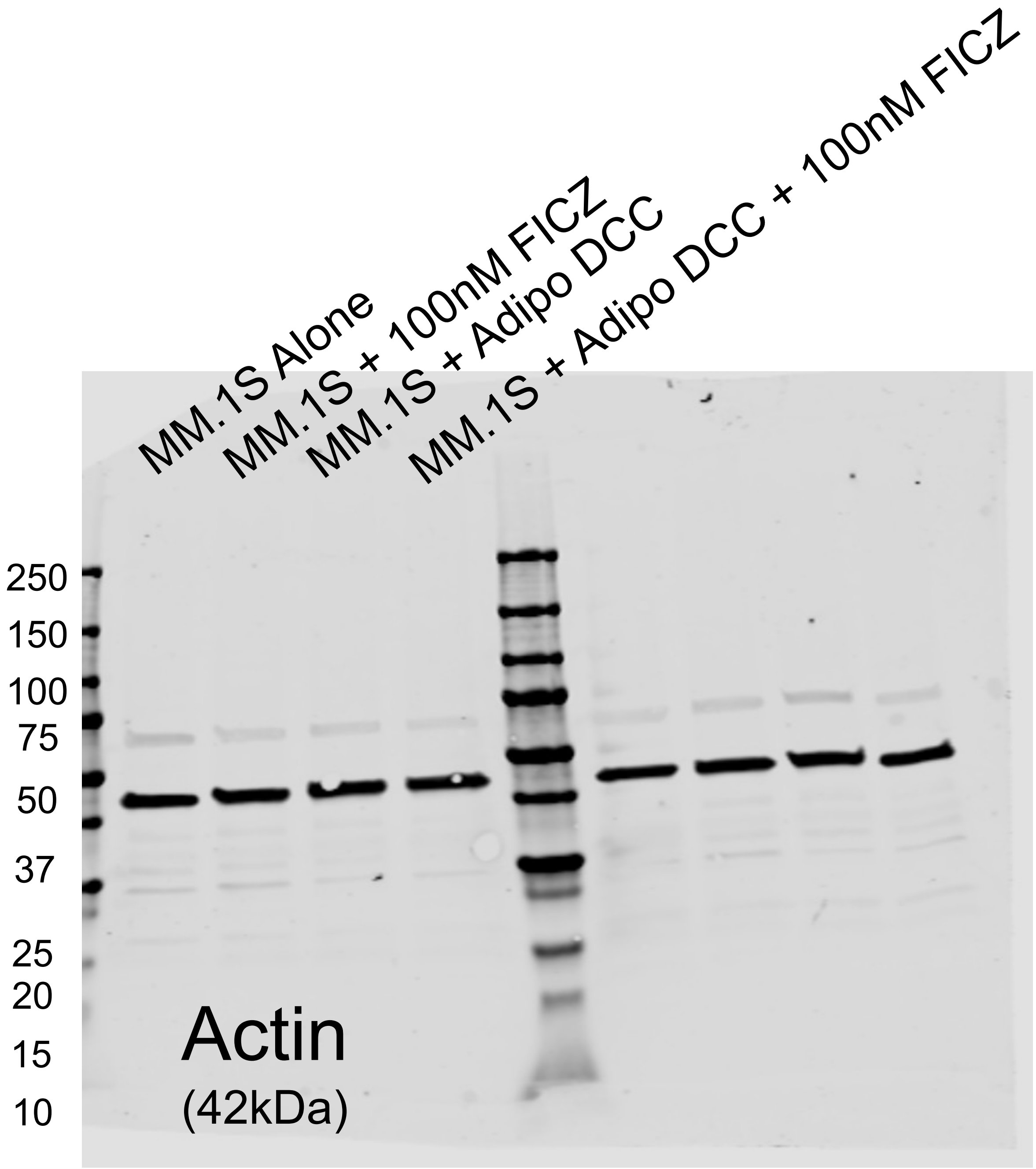

Figure 5A –
MM.1S Alone vs Adipo CM

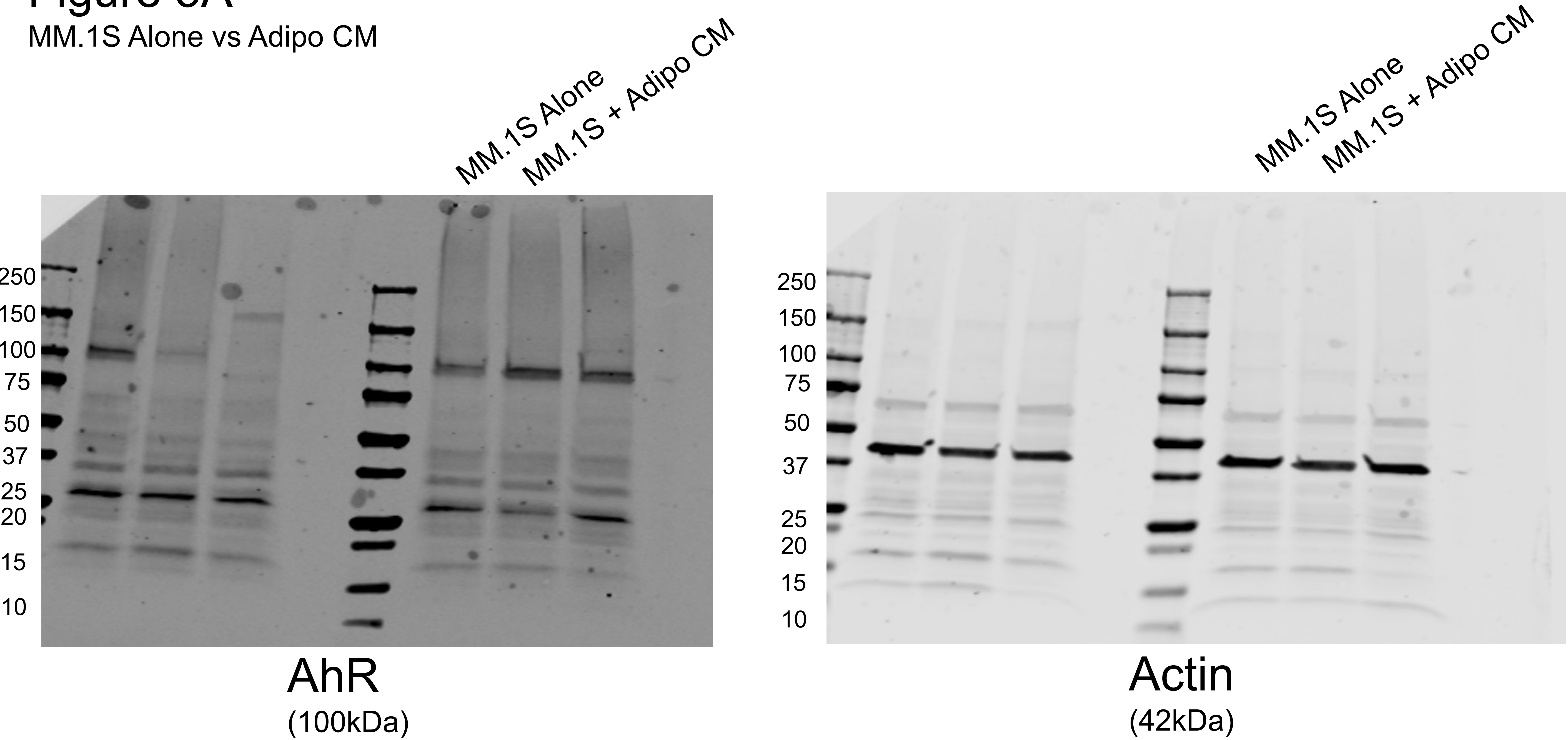

Figure 5C –

MM.1S AhR KD and EV

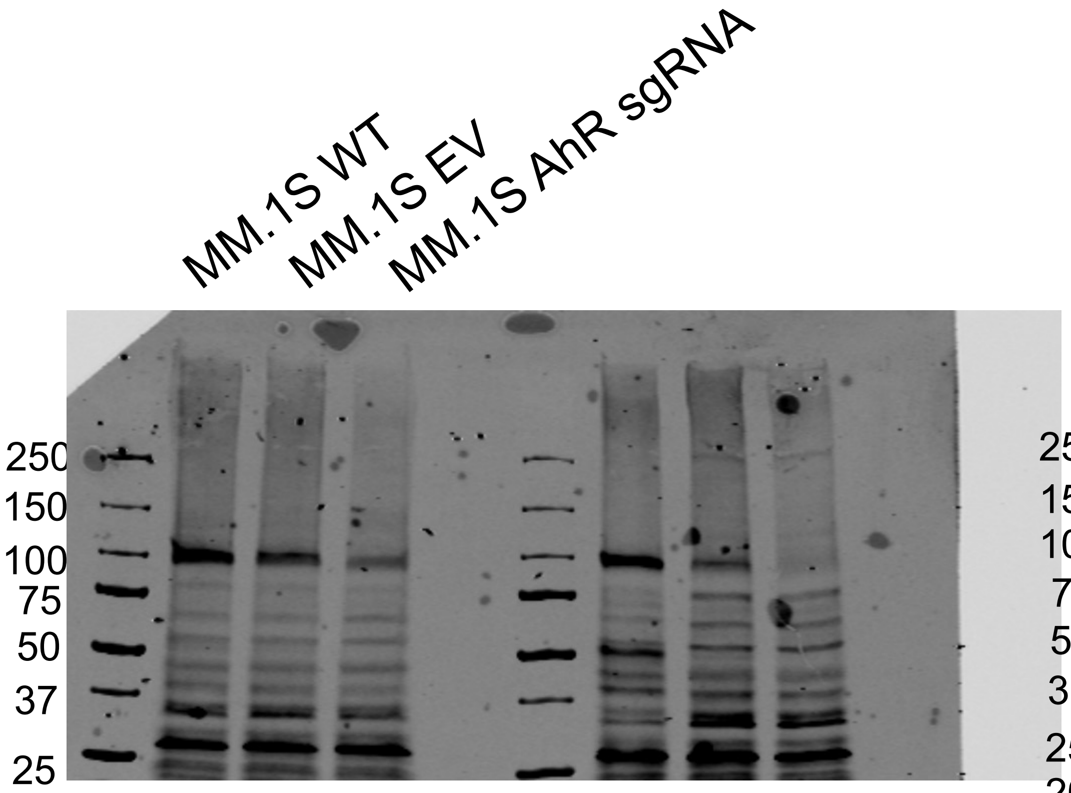

AhR
(100kDa)

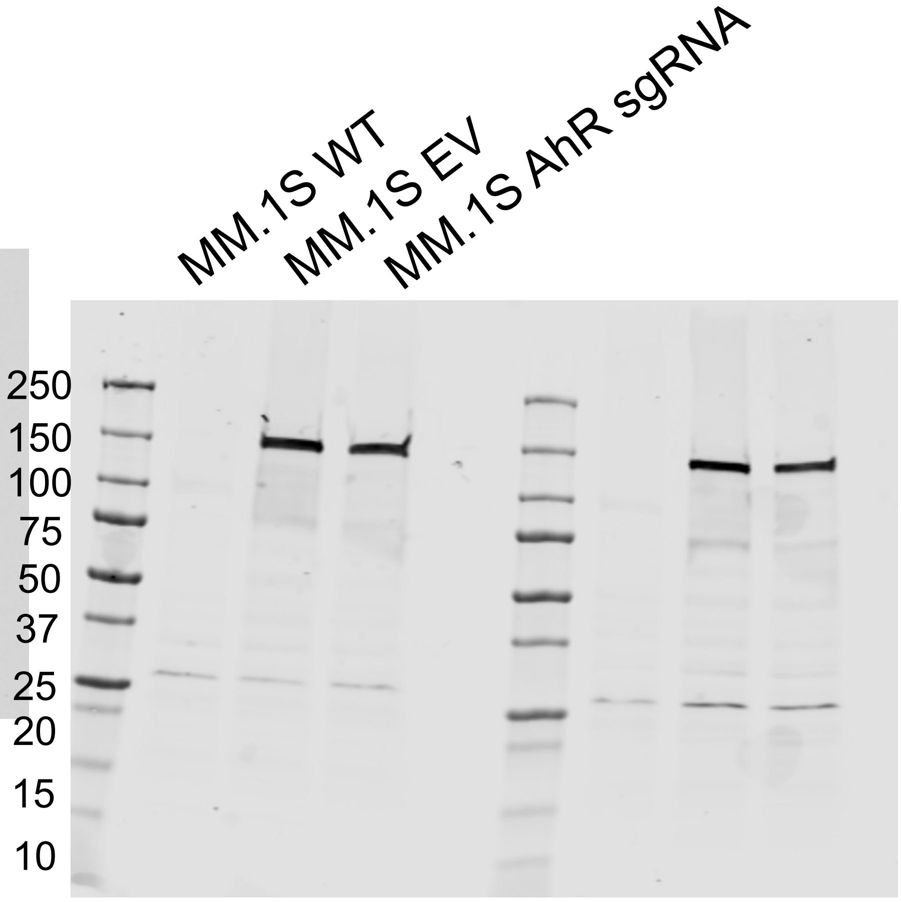

Cas9
(160kDa)

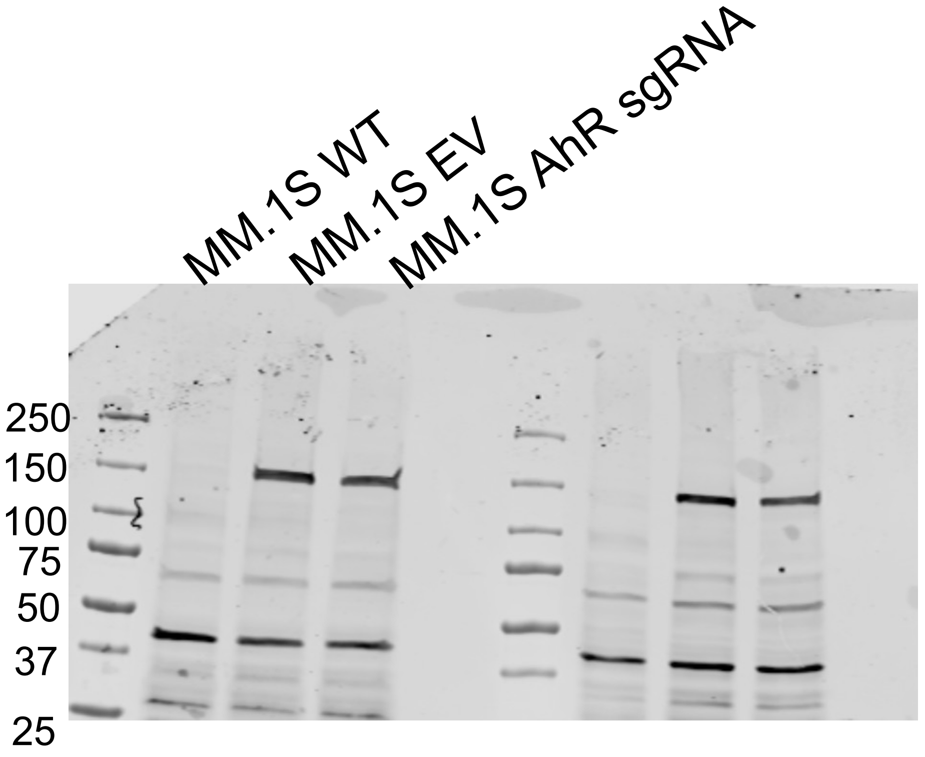

Actin
(42kDa)
