# Supplementary material for: Non-Toxicological Role of Aryl Hydrocarbon Receptor in Obesity-Associated Multiple Myeloma Cell Growth and Survival
Source: Cancers (Basel). 2023 Nov 1;15(21):5255. doi: 10.3390/cancers15215255 (PMC10649826; doi:10.3390/cancers15215255)
Supplement: Supplementary file 1 [file cancers-15-05255-s001.zip › supplementary material/cancers-2634977-supplementary.docx]

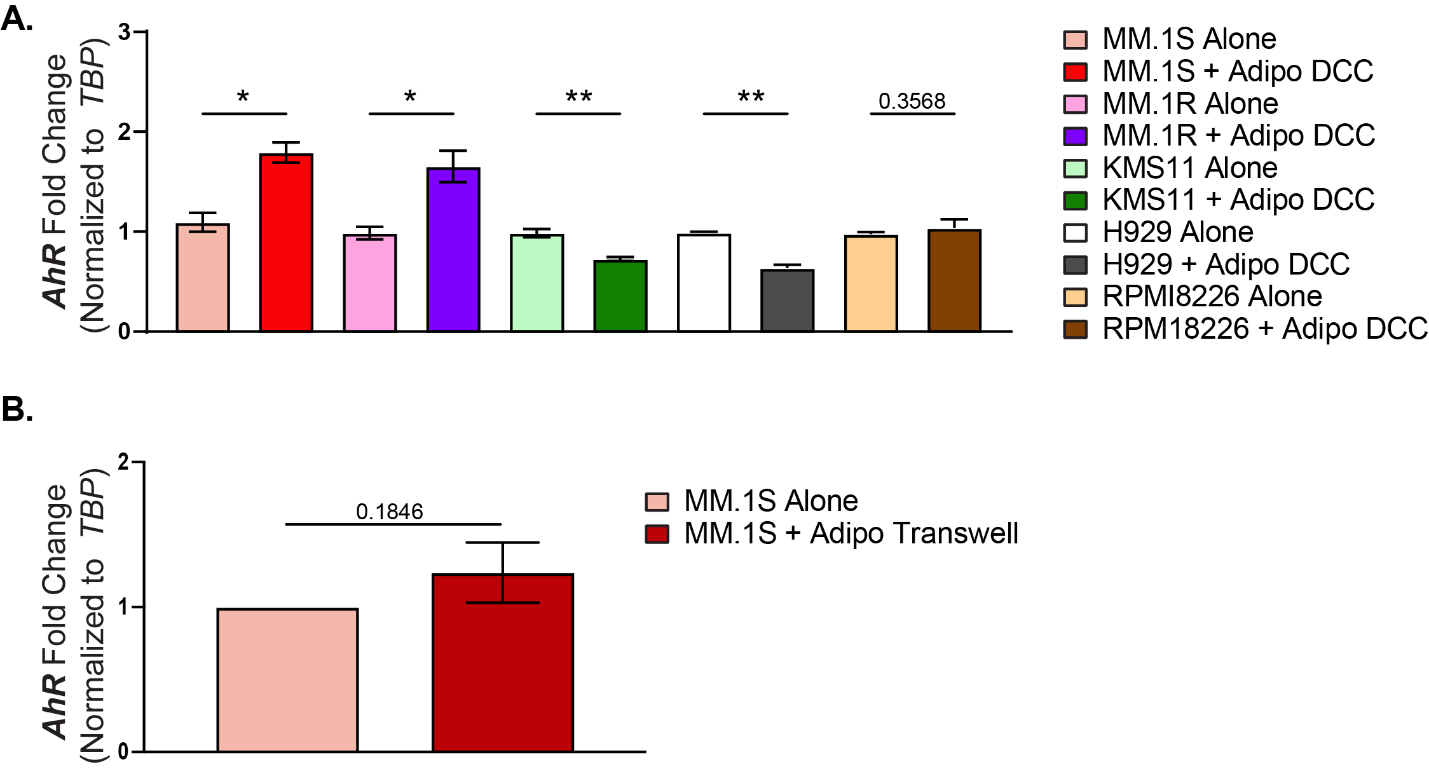


**Supplemental Figure 1: AhR gene expression does not mimic protein levels in response to adipocyte co-culture or transwell co-culture.** **A.** Taqman qPCR analysis of AhR levels in the human MM cells grown alone or direct co-culture with adipocytes (Adipo DCC). Individual t-tests were used to determine significance relative to cells grown alone. **B.** Taqman qPCR analysis of AhR expression in MM.1S cells grown alone or in transwell co-culture with bone marrow adipocytes (Adipo Transwell). Significance values are depicted as p < 0.05 = *, p < 0.01 = **. P values that failed to reach significance are displayed.


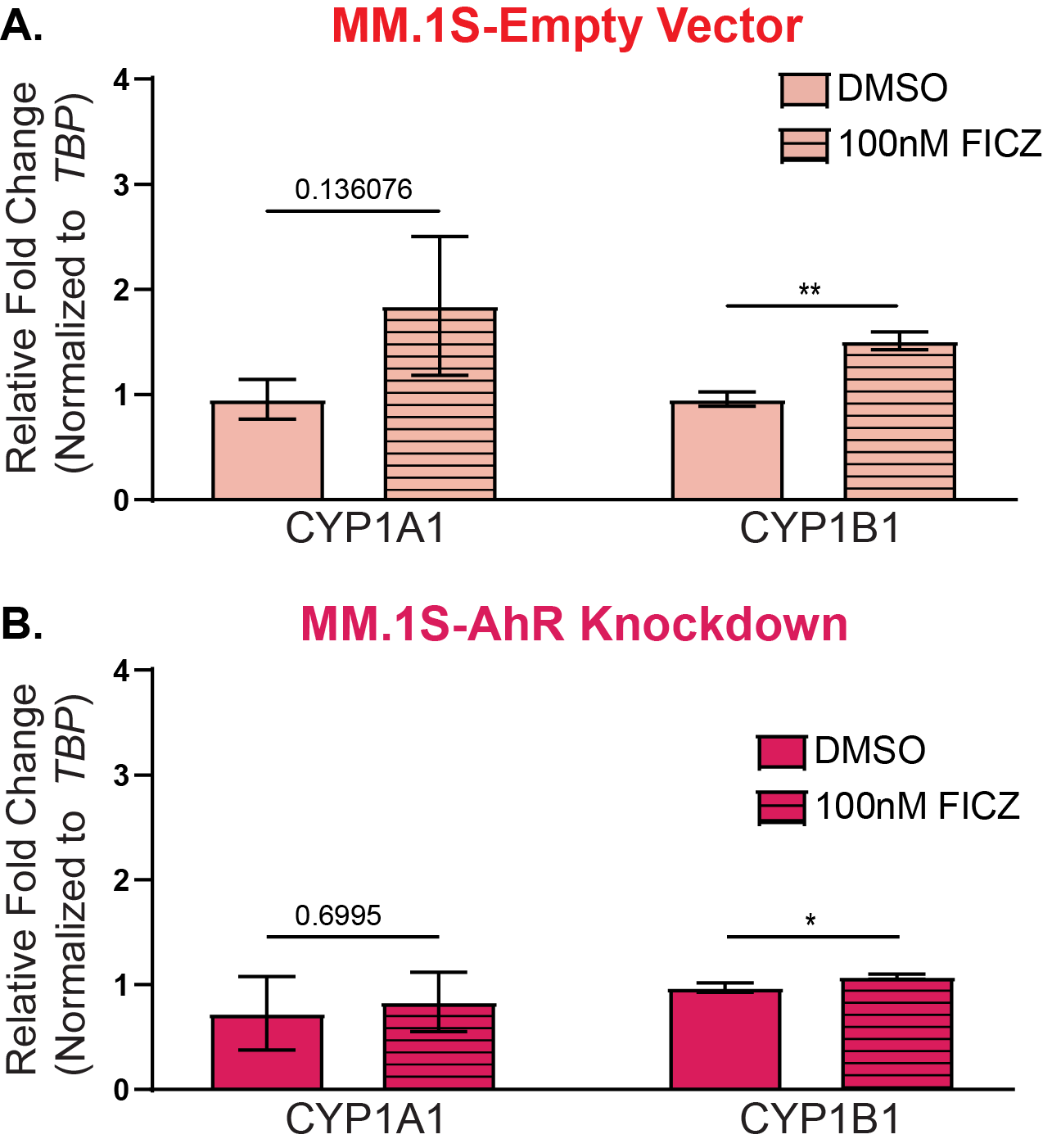


**Supplemental Figure 2: MM.1S-AhR Knockdown cells do not respond to FICZ.** Taqman qPCR analysis of CYP1A1 and CYP1B1 gene expression in MM.1S-Empty Vector (**A.**) and AhR Knockdown (**B.**) cells in the presence of absence of FICZ (100nM). CYP expression was normalized to TBP expression and results are shown as a fold change relative to their vehicle control treated cells. Individual t-tests were used to determine significance relative to the vehicle control cells and significance values are depicted as p < 0.05 = *, p < 0.01 = **. P-values that fail to reach significance are displayed.


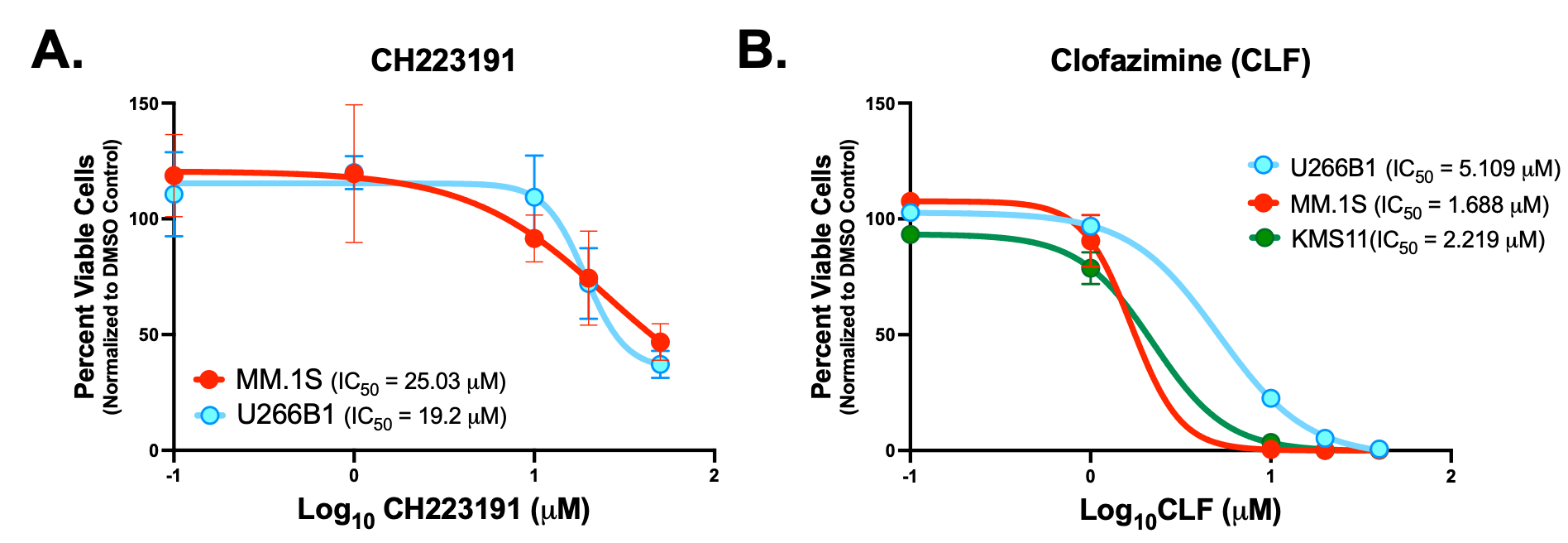


**Supplemental Figure 3: AhR antagonists are cytotoxic to MM cells independent of AhR status.** CellTiter-Glo viability assay of MM.1S and U266B1 cells in response to AhR antagonist CH223191 (**A.**) and Clofazimine (CLF) with the addition of KMS11 cells (**B.**) for 72 hours. All values are represented as a percentage of viable cells normalized to the vehicle control and IC_50_s were determined using GraphPad Prism.
